# A Comprehensive Review on Solitary Fibrous Tumor: New Insights for New Horizons

**DOI:** 10.3390/cancers13122913

**Published:** 2021-06-10

**Authors:** Javier Martin-Broto, Jose L. Mondaza-Hernandez, David S. Moura, Nadia Hindi

**Affiliations:** 1 Fundacion Jimenez Díaz University Hospital, 28040 Madrid, Spain; nhindi@atbsarc.org; 2 General de Villalba University Hospital, Collado Villalba, 28400 Madrid, Spain; 3 Fundación Jiménez Díaz Institute for Medical Research (IIS/FJD), 28040 Madrid, Spain; 4 Institute of Biomedicine of Seville (IBiS, CSIC, US and HUVR), 41013 Sevilla, Spain; Jose.mondaza@quironsalud.es (J.L.M.-H.); david.moura@usal.es (D.S.M.)

**Keywords:** solitary fibrous tumor, therapy, anti-angiogenics, tumor biology

## Abstract

**Simple Summary:**

Solitary fibrous tumor (SFT) is a malignant condition that exhibits different clinical behaviors ranging from low to high aggressive SFT, with dedifferentiated SFT (DD-SFT) being the fastest-growing subtype. Even when surgery alone provides curation rates above 60%, recurrences do occur in a fraction of patients where surgery is unable to provide disease control. Among the systemic therapeutic options, antiangiogenic compounds have shown higher efficacy than chemotherapy by indirect comparisons. Furthermore, rotating different antiangiogenics, at the progression time, has been shown to be effective. The exception is DD-SFT since it is resistant to antiangiogenics but can respond to chemotherapy. This comprehensive review also analyzes the underlying molecular components that play a key role in SFT origin and aggressiveness. The discovery in 2013 of anomalous fusion genes between *NAB2* and *STAT6* was determinant to increase the knowledge on the molecular drivers in SFT that could be potential targets for future therapies.

**Abstract:**

Solitary fibrous tumor (SFT) is a rare mesenchymal, ubiquitous tumor, with an incidence of 1 new case/million people/year. In the 2020 WHO classification, risk stratification models were recommended as a better tool to determine prognosis in SFT, to the detriment of “typical” or “malignant” classic terms. The risk for metastasis is up to 35–45%, or even greater, in series with a longer follow-up. Over the last few decades, advances in immunohistochemistry and molecular diagnostics identified STAT6 nuclear protein expression and the *NAB2–STAT6* fusion gene as more precise tools for SFT diagnosis. Recent evidence taken from retrospective series and from two prospective phase II clinical trials showed that antiangiogenics are active and their sequential use from first line should be considered, except for dedifferentiated SFT for which chemotherapy is the best option. Since the fusion transcript driver’s first description in 2013, new insights have been brought on key molecular events in SFT. This comprehensive review mainly focuses on the superior efficacy of antiangiogenics over chemotherapeutic agents in SFT, provides the current knowledge of key molecules that could co-drive the SFT behavior, and suggests new target candidates that deserve to be explored in preclinical and clinical research in SFT.

## 1. Introduction

Solitary fibrous tumor (SFT) has an age-adjusted incidence rate of 0.61 and 0.37 per million persons per year for extrameningeal [1] and meningeal [2] cases, respectively; in other words, we can assume an age-adjusted yearly incidence of 1 new case per million people. The 2013 WHO classification integrated under the SFT nomenclature the former hemangiopericytoma denomination, [3] and the 2020 WHO classification eluded the terms of “typical” or “malignant” as typical SFT was not necessarily synonymous with benign disease [4]. Instead, risk stratification models were recommended as a better tool to determine prognosis in SFT. The risk of metastatic spread can be as high as 35–45%, or even greater, in series with a longer follow-up period [5,6]. One of these series reported a 5-year metastasis-free rate of 74%, while for the 10-year metastasis-free rate, this figure decreased by 55%. Recurrences beyond 10 years were seen in up to 10% of the SFT patients [7]. Even with a longer follow-up, the relapse-free survival can be as low as 18% at 20 years [8].

In recent years, the diagnostic and therapeutic landscape of patients with solitary fibrous tumor has been refined and several antiangiogenic drugs have been tested in this context, and also in a prospective setting, constituting the drugs with the greatest evidence of activity on this entity. Other therapeutic strategies, such as surgery (the cornerstone in localized disease) or radiotherapy, are not the main aim of this paper. This review will focus on the underlying biological processes that can explain paraneoplastic syndromes or histological phenotypes or related signaling pathways to fusion transcript that could explain the superior efficacy of antiangiogenics over chemotherapeutic agents detected in SFT. To this end, this comprehensive review will bring insights into possible mechanisms, with a deep dissection of the molecular context.

## 2. Clinical Aspects

SFT is considered a fibroblastic tumor with ubiquitous allocation affecting adult patients, usually from 20 to 70 years. In the largest series, the extrameningeal SFT cases were distributed as follows: abdominal cavity 31%, limbs 29%, pleura 22%, trunk 11% and others 7% (including head and neck but not meninges) [9]. The median age reported in the largest series ranged from 50 to 60 years [6,9]. Clinically, SFTs present as a well-defined mass, which is more silent in primary pleural locations than extra-pleural primary sites.

### 2.1. Paraneoplastic Syndromes

Few patients, less than 10%, suffer paraneoplastic syndromes such as hypertrophic osteoarthropathy (HOA), seen in cases of pleural SFT. HOA syndrome is more frequent than another reported paraneoplastic syndrome (hypoglycemia), and it could be underestimated in SFT. The overexpression of vascular endothelial growth factor (VEGF) is deemed to be the main underlying pathogenic mechanism (see below). HOA usually includes finger clubbing, hypertrophic skin changes, arthralgia and increased periosteal activity. Nevertheless, distal digital clubbing could be presented as an asymptomatic condition, but when clubbing is associated with pain, HOA is usually the underlying process. Inspection of clubbing in distal phalanges (hands or feet) can uncover an underlying increase in vascularization (floating nail sign when proximal nail bed is slightly pressured). In addition, in clubbing fingers, the distal phalangeal depth is larger than the distal interphalangeal depth. This dimension is measured from the side [10]. The diagnosis of HOA is based on the combination of clubbing and an increase in periosteal activity detected in plain X-ray. Currently, it is unclear if clubbing and HOA are different expressions of the same entity or if they are different conditions with different pathophysiology. Megakaryocytic hypothesis is widely accepted as the main pivotal cause for clubbing. Megakaryocytes would reach systemic circulation from pulmonary disrupted circulation. This latter can be induced by aberrant vessel formation, as in the case of pleural SFT. A minor fraction of such megakaryocytes would reach the distal capillaries. After impaction, megakaryocytes and thrombocytes secrete platelet-derived growth factor (PDGF) and VEGF that can induce the mesenchymal changes seen in clubbing fingers. The larger distance would explain why toes are less frequently affected than fingers, as the probability of megakaryocytes reaching the distal capillaries of toes is lower. Histologic findings in clubbed fingers, such as the presence of platelet aggregates in capillaries as well as the overexpression of *VEGF* and *PDGF* in these aggregates, pericytes and stromal fibroblasts have been detected and are in line with the hypothesis [11,12]. Systemic VEGF secretion is demonstrated in the serum of patients with lung cancer with HOA in comparison to patients with lung cancers without HOA. Moreover, a drastic resolution of skeletal changes and VEGF plasmatic levels have been detected after tumor resection [13]. This overexpression of VEGF could potentiate the local production mentioned for clubbing and could explain the transforming findings seen beyond distant fingers/toes, such as in the tongue or skin for instance [10,11]. Changes in the latter can even be confounded with acromegalic changes, but in HOA the growth hormone could be decreased rather than increased. Another reported paraneoplastic syndrome is hypoglycemia, seen in less than 5% of SFT cases. This is called non-islet cell tumor hypoglycemia and its pathogenesis is related to the overproduction of insulin-like growth factor-II [14]. Other findings include low levels of blood glucose, low values for growth hormone and low levels of insulin-like growth factor-I or III.

### 2.2. Radiological Characteristics

Radiologically, tumors were considerably vascular, showing an avid contrast enhancement in 65% of cases in CT scans and, interestingly, in 35% of cases large collateral feeding vessels were verified [15]. Heterogeneity after contrast injection was more frequently seen in more aggressive than in more indolent SFTs, 76.5% vs. 40.0% [16]. SFT exhibits intermediate to high attenuation on unenhanced CT scans, reflecting a high density of collagen fibers along with a rich capillary network [17]. In other series, heterogeneous attenuation was detected in 88% of cases. Low attenuation on unenhanced CT scans could be present as geographic, focal or linear disposition and is observed in up to 86%. These low attenuated areas correspond with gross necrosis, hemorrhage or cystic changes. In MRI, SFT appears as isointense in T1 weighted images and variable in T2. Low intensity areas on T1 or T2 weighted images are due to collagen content and low cellularity. Strong enhancement with gadolinium is usually seen as it is consistent with vascular tumors [18]. The ^18^F-FDG PET has not been shown to be a determinant in distinguishing indolent SFT (the old fashioned typical SFT) from aggressive SFT (the old-fashioned malignant SFT) in a series of 17 patients with confirmed SFT diagnosis [19]. Obviously, different metabolic activity could be detected in extreme cases, [18] especially if dedifferentiated SFT is considered. However, it is unclear if mild overproduction of insulin-like growth factor II could have some influence on the PET scan.

### 2.3. Risk Classification

The last WHO classification appropriately avoids the terms “typical” and “malignant” in the context of SFT, since it can be misleading to identify a “typical” subtype with a benign condition [20]. It is true that once “low aggressive SFT” (formerly “typical”) was diagnosed, the biological behavior, even in a metastatic setting, was more indolent [21]. In this way, it could make sense to distinguish three different subsets of SFT: low aggressive, high aggressive and dedifferentiated SFT, even when all of them represent different parts of a continuous spectrum. Nevertheless, a pragmatic approach is to estimate the risk of recurrence in every newly diagnosed SFT. Several risk classification models have been reported for localized resected extrameningeal SFT (Table 1) [6,9,22,23,24,25]. Among these, two have been externally validated and therefore offer a more accurate prediction for the risk of metastasis [6,9,24,26]. One is based on age, size, mitotic count and tumor necrosis and distributes the patients into three different risk categories. Another risk model estimates the individual risk for local and metastatic recurrence. This latter probability was calculated scoring for age, mitotic count, and tumor localization (limb vs. others).

## 3. Pathogenesis and Pathology

### 3.1. Morphological Features

**Figure 1:**
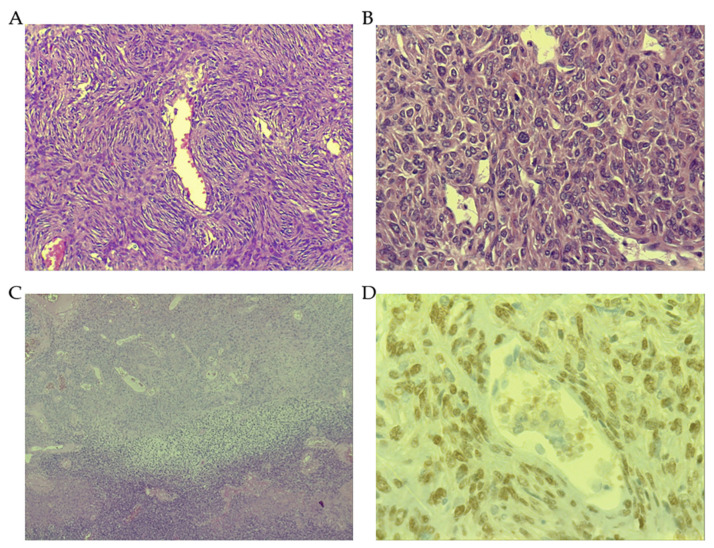
Histopathological features of solitary fibrous tumor (SFT). (**A**) Low-grade SFT showing a patternless pattern, with spindle cells, low number of mitosis (or lack of) and vessels with “staghorn” appearance (Magnification 40×). (**B**) High-grade SFT showing hypercellularity with nuclear pleomorphism, high number of mitotic figures (Magnification 200×). (**C**) Dedifferentiated SFT with an abrupt transition from conventional SFT to high-grade sarcoma (Magnification 10×). (**D**) STAT6 positive nuclear immunostaining (Magnification 400×).

An SFT is comprised of randomly arranged cells with spindle or ovoid shape, within a collagenous stroma, intermixed with blood vessels with a characteristic staghorn shape. The disposition of cellular and stromal components in SFT is called “patternless pattern” and the intercellular collagen bands, if we may be so bold, could be said to evoke Van Gogh lines. The histological spectrum goes from a paucicellular context with abundant stromal collagen to highly cellular tumors where stroma is hardly detected. The mitotic count is more frequently low, and it is crucial for establishing a recurrence risk. Other pathologic findings that could have some influence on recurrence risk are nuclear pleomorphism, necrosis and cellularity. Fat-forming SFT carries a component of mature adipose tissue, and it is more frequently seen in the context of more indolent SFT. Yet, some aggressive SFTs have been also described with this feature, where the presence of lipoblasts and an atypical lipomatous tumor is seen with higher frequency than in low risk or more indolent SFTs [28]. Giant cell-rich SFT, formerly known as giant cell angiofibroma, shows features of SFT admixed with multinucleated giant cells within the stroma [29]. This characteristic SFT is more frequently, but not exclusively, found in the head and neck region. Dedifferentiated SFT constitutes the most aggressive SFT subtype. Histologically, it exhibits an abrupt transition zone to high-grade sarcoma that could contain heterologous elements such as, for instance, rhabdomyosarcoma or osteosarcoma (Figure 1) [30].

### 3.2. The Role of Signal Transducer and Activator of Transcription 6 (STAT6)

Immunohistochemically, strong nuclear staining of signal transducer and activator of transcription 6 (STAT6) has become characteristic of SFT. As explained below, the underlying transcript between NGFI-A binding protein 2 (EGR1 binding protein 2) (*NAB2*) and *STAT6* is formed after the replacement of at least one repressor domain of *NAB2* with a transactivation domain of *STAT6*, which results in an overexpression of EGR1, a main target of the *NAB2* gene. The *STAT6* gene encodes a cytoplasmic protein (in a continuous shuttling to the nucleus), which acts as a transcription factor, in contrast to *NAB2*, that normally functions as a transcriptional repressor through its interaction with EGR1 and is localized in the nucleus. The transcript *NAB2–STAT6* acts as transcriptional activator, inducing the expression of EGR1 target genes. Interestingly, the transcript translocates to the nucleus resulting in a high level of expression of the transcript (*NAB2* is also a target of EGR1) compared with other tumors or normal tissues. This is the rationale for the characteristic nuclear STAT6 immunostaining, which is tremendously helpful for the diagnosis of SFT [31]. Of note, in the presence of a higher genomic instability and cell reprogramming observed in dedifferentiated SFT, the expression of oncoprotein transcript *NAB2–STAT6* can be lost [32]. It is unclear whether *STAT6* acts as a key player in the pathogenesis of SFT or rather plays a secondary role. Intriguingly, *STAT6* can induce fibrosis in the interactive network context of wound healing between macrophages, CD4 (Th2) and fibroblasts, signalizing through IL-13 [33]. As this latter cytokine signal by *STAT6*, a nexus with EGR1 and PDGF has been established in the pulmonary context, in the subepithelial fibrosis of distal airways [34]. However, these IL-13 mediated actions have been described in the context of wild-type *STAT6*, hence it is doubtful whether this is displayed in SFT in the same way. In any case, it seems judicious to consider STAT6 as a potential key protein in the SFT pathogenesis as well. The fact that EGR1 is target of both *NAB2* as a transcriptional repressor and *STAT6* as a transcription factor results in an interesting meeting point even when something other than the canonical IL-13/STAT6 pathway, which induces phosphorylation and posterior dimerization of STAT6 to enter in the nucleus, will be required. Further, as *NAB2* and *STAT6* are involved in the regulation of inflammation, collagen production, fibroblast activation and vessel formation, a deeper microenvironment study is needed in SFT to deconvolute the mechanisms due to each one.

Besides, strong nuclear staining for STAT6 has been observed in other mesenchymal tumors (Table 2), including well-differentiated and dedifferentiated liposarcoma (WD/DD-LPS) [35]. In the latter, STAT6 overexpression could be related to the amplicon intricately linked to WD/DD-LPS pathogenesis given the fact that *STAT6* is located at chromosomal region 12q13 [36]. In all of these tumors, the strong nuclear immunostaining of STAT6 concurrently occurred with strong cytoplasmic expression, indicating a higher than normal shuttling of STAT6 between the cytoplasm and nucleus.

### 3.3. Other Immunohistochemical Markers

Other unspecific supportive immunostaining markers used in SFT diagnosis are CD34, bcl-2 and CD99. The expression of CD34 is strong and diffuse in more than 80% of SFT tumors, but its expression can be lost in the most aggressive SFTs [37,38]. CD34 is a glycoprotein of the cellular membrane, which is expressed in several normal tissues: hematopoietic precursors, endothelial, endoneurial or fibroblastic cells (Table 2). Interestingly, several low-grade fibroblastic and myofibroblastic tumors apart from SFT can also show diffuse and strong CD34 immunostaining (Table 2).

Even when the molecular structure of CD34 is well recognized, its function is far from being completely understood. In hematopoiesis, CD34 has roles of cytoadhesion and the regulation of cell differentiation. CD34^+^ cells represent a proportion of the total mesenchymal stem cells (MSCs), and are associated with high colony forming efficiency and long-term proliferative capacity [39,40]. Moreover, CD34^+^ MSCs have exhibited a propensity for endothelial transdifferentiation. Thus, CD34^+^/CD90^+^ cells of human adipose tissue were able to form a sphere cluster and be differentiated in endothelial cells that form capillary-like structures producing a high level of VEGF [41]. A diffuse cytoplasmic with perinuclear enhancement of bcl-2 staining has been constantly described in SFT [42]. The level of bcl-2 positivity ranges from 70 to 86% in the largest series of SFT [37,43]. Of note, bcl-2 immunostaining was positive in benign conditions with spindle cellularity (Table 2). Further, apart from SFT, it is expressed in some fibroblastic spindle cell sarcomas and in the spindle component of DD-LPS or in synovial sarcoma. A close relationship of CD34 staining is seen with bcl-2, being coincident in several tumors such as SFT, dermatofibrosarcoma protuberans, Kaposi sarcoma or gastro-intestinal stromal tumor (GIST), as well as in other previously mentioned benign conditions [44]. In SFT, bcl-2 expression was seen regardless of the mitotic activity or the cellularity. Analyses in mammalian tissues determined that bcl-2 protein expression is common in stem cells, endocrine tissue, and long-lived cells [45]. Considered together, this raises the possibility that the pathogenesis of SFT could be explained as a result of neoplastic transformation of a fibroblastic precursor CD34^+^/bcl-2^+^. In addition, bcl-2 expression could be induced by STAT6 through IL-4. This mechanism is physiologically activated in lymphocytes where this signaling pathway would maintain the T cells activated, avoiding apoptosis [46]. The overexpression of bcl-2 in SFT could explain the chemo-resistance seen in this entity. Additionally, the bcl-2 expression detected in synovial sarcoma could be explained by the characteristic translocation t (X, 18) that would affect the bcl-2 gene allocated in chromosome 18 [47]. The positive expression of bcl-2 seen in neural neoplasms could be due to the fact that these tumors stem from the neural crest cell lineage, which also expresses bcl-2. The protein expression of CD99 is extensively present in SFT, showing strong membranous predominant staining, or membranous cytoplasmic, in more than 80% of cases. The glycosylated transmembrane protein CD99 is implicated in several cellular functions such as cell adhesion, migration, differentiation, endo and exocytosis among others [48]. In malignancy, CD99 has been demonstrated to have a remarkable role in migration, invasion and metastasis. In this sense, CD99 has behaved as an oncogene in several tumors including some sarcomas such as Ewing sarcoma, synovial sarcoma or rhabdomyosarcoma. However, there is an increasing number of tumors in which CD99 expression is diffuse in an early stage or in benign conditions, but is lacking or reduced in an advanced stage or in the malignant counterpart [49,50]. In this latter subset of tumors, which includes osteosarcoma, CD99 acts as a suppressor gene. In addition, two isoforms of CD99 (wild type and truncated forms) have been described with opposite functions. While CD99^wt^ inhibits migration, metastasis, anoikis resistance and anchorage-independent growth, the truncated form exhibits the opposite functions [51]. CD99 is highly expressed in CD34^+^ bone marrow cells and in leukocytes, whatever the lineage, and it is a determinant in the orientation of immune response [52]. As CD99 expression is usually lost in the dedifferentiated SFT (in the DD zones), it is probable that CD99 would act also as a tumor suppressor in the context of SFT [53].

In all, one might speculate that precursor cells of SFT would harbor an early expression of CD34+ and bcl-2, probably a progenitor of fibroblasts or myofibroblasts, along with CD99+ upon which other genetic early hits had been added, such as the characteristic NAB2–STAT6 transcript. The latter would ultimately facilitate proliferation through EGR1 signaling.

## 4. Dedifferentiated SFT (DD-SFT)

Dedifferentiation can occur at the end of the transforming histological stage of SFT, reflecting that new genetic hits have emerged in the tumor or that dedifferentiated clones have evolved from the initial malignant process, until they govern the tumor biology. This dedifferentiation process is not exclusive to SFT, rather it is observed in a wide spectrum of malignancies, such as melanomas, carcinomas or even other sarcomas (DD-LPS or chondrosarcomas, for instance). This subtype can be underestimated when core biopsies do not reach the dedifferentiated component or after a resection of bulky SFT if the sampling is not complete. DD-SFT can be diagnosed de novo or following a recurrence of indolent or aggressive SFT. Characteristically, DD-SFT is diagnosed if an abrupt area of high grade sarcomatous or anaplastic cells appears in the SFT bed. As previously mentioned, some protein expressions are frequently lost in DD-SFT, such as CD34, [38,54,55] CD99 [56] and STAT6 [32,57]. It is not yet clear which are the mechanisms underlying the loss of expression of the previous proteins, but a kind of post-translational control through ubiquitination has been postulated, at least for the loss of STAT6 nuclear expression [32]. The mutation of *TP53*, and accordingly, a nuclear positive immunostaining, has been found in some high-grade SFTs and in many DD-SFTs. This finding was reported a long time ago [38,58] and corroborated in recent times by comparative genomic hybridization, demonstrating the loss of 17p, always involving *TP53,* in high-grade and DD-SFT [32,59,60]. Another onco-suppressor, *RB1*, is also frequently lost in DD-SFT, something supported by the disappearance of previously verified nuclear immunostaining and by the most frequent copy number abnormalities, the loss of 13q, always affecting *RB1* [32]. Interestingly, in the transit towards higher dedifferentiation, SFT cells display a complex cytogenetic profile with numerous copy number alterations, indicating an increase in genomic instability [32]. Oxidative stress (ROS) could also contribute to this instability via EGR1, which is a transcriptional activator of *NOX4*. In some disease contexts, such as diabetic kidney disease, *EGR1, NOX4* and *ROS* have been found as critical components [61]. Interestingly, *NOX4* is overexpressed in SFT. These genomic gains and losses are nonrandom events, likewise other dedifferentiation processes observed in sarcoma, such as DD-LPS or DD chondrosarcoma, where recurrent genomic events are detected. The critical genomic events that induce different SFT subtypes from one precursor or maybe convert low-grade SFT into high-grade SFT and then to DD-SFT, changing from *NAB2–STAT6* addiction to other genomic drivers, are just being explored from recent times.

Insulin-like growth factor 2 (IGF2) and insulin-like growth factor 2 receptor (IGF2R) are overexpressed in a substantial proportion of SFT cases, and this overexpression is detected by immunohistochemistry [62]. In fact, the serum increase in IGF2 would be responsible for the hypoglycemic syndrome (Doege–Potter syndrome). Hypoglycemia has been proposed as an independent prognostic marker in SFT for a higher probability of metastatic recurrence and death [63]. Along the same lines, early reports already related the appearance of hypoglycemia to a larger tumor diameter and more aggressive behavior [64]. However, some controversies still remain regarding the prognostic implications and functionality of IGF2R. Additionally, the overexpression of interferon-stimulated gene 15 (*ISG15*) significantly correlated with worse PFS and OS in translational research of a phase II clinical trial exploring pazopanib as first line of antiangiogenesis in advanced/metastatic SFT [65]. *ISG15* has been implicated in stemness, cancer survival and drug resistance [66].

The presence of *TERT* promoter mutation had a worse prognostic role in some series [67,68] but not in others [69]. Therefore, further investigation is needed to know its real prognostic impact.

## 5. Molecular Biology

**Figure 2:**
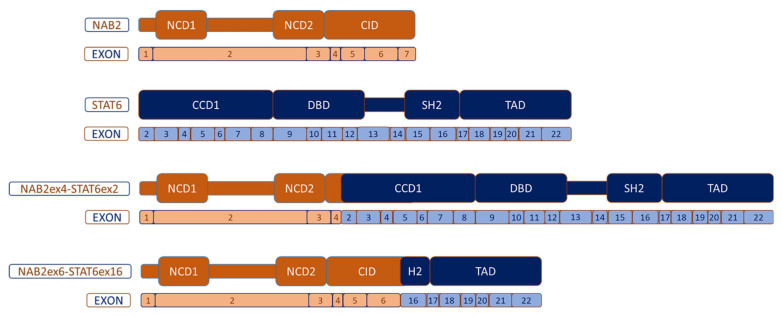
Most common *NAB2–STAT6* fusion variants. NCD: NAB-conserved domain; CID: CHD4-interacting domain; CCD1: Coiled-coil domain 1; DBD: DNA-binding domain; SH2: Src homology 2 and TAD: transcriptional activator domain.

The identification of *NAB2–STAT6* fusion within chromosome 12, communicated in 2013 by three different research groups, was an important milestone in terms of understanding this entity [70,71,72]. According to some authors, the key dysfunction in the transcript *NAB2–STAT6* lies in the disturbed function of *NAB2*, rather than *STAT6* deregulation. The reason is based on the fact that all the genomic fusions between *NAB2–STAT6* entail a protein transcript that exchanged at least one repressor domain from NAB2 for an activation domain from STAT6. This results in a dysregulation of early growth response (EGR) signaling. NAB2 is constituted, on the one hand, by an N-terminal binding domain (NAB2 conserved domain 1-NCD1) that interacts with EGR1, and NCD2 which is important for transcriptional repression and, on the other hand, the carboxy-terminal, which includes a chromodomain helicase DNA-binding protein 4 (CHD4) interacting domain (CID), important for transcriptional repression as well. CHD4 is a subunit of the nucleosome remodeling and histone deacetylase complex (NuRD) with enzymatic function (Figure 2).

As the EGR1-interacting NCD1 is always present in the fusion protein, an enhancement of the expression of EGR1-target genes is expected in SFT. EGR1 is one of four cysteine-rich zinc finger transcription factors. *EGR1* expression is activated by different factors such as cytokines, hormones or growth factors such as TGF-β. This gene has been implicated in cancer, inflammation or fibrosis. Of note, *NAB2* is a target of EGR1 and *EGR1* is a target of NAB2. This latter regulation is produced by the binding of ERG1 to the R1 inhibitory domain and through CHD3 and CHD4 proteins that have enzymatic function and belong to NuRD, a remodeling chromatin complex with relevance in transcription regulation [73]. As the wild type *NAB2* gene is a repressor of EGR1 transcription activity, and in the context of the *NAB2–STAT6* transcript, where the carboxi-terminal parts of *NAB2* (relevant for transcriptional repression) have been exchanged, an increase in transcription for *EGR1* target genes could be expected. However, this was not found in some investigations [72]. This could be explained by the fact that *NAB2* sometimes potentiates rather than represses *EGR1* transcription. In any case, EGR1′s effects are not fully characterized. Among those EGR1 target genes found to be dysregulated in SFT, several are involved in fetal development. Thus, *HOX* genes (class I homeobox genes) have been shown to be overexpressed in SFT, except for *HOXD* [71,72].

Of note, NuRD also has a relevant prominence for the normal differentiation of embryonic stem cells by downregulating the expression of *ZFP42*, *TBX3*, *KLF4*, and *KLF5* genes. Intriguingly, some of these genes are downregulated in SFT [72]. In other words, in the context of SFT, NuRD’s function is to restrict these pluripotency genes. It should be questioned if in DD-SFT some of the NuRD complex functions are lost, such as the *Mbd3*, since the mutation of this gene induced the overexpression of the pluripotency genes mentioned above [74].

On the other hand, the *STAT6* gene is a member of the *STAT* family encoding cytoplasmic transcription factors, which regulate gene expression, transmitting signals to the nucleus and binding to certain DNA promoters. The *STAT6* gene consists of 23 exons and functionally has the following structure: N-terminal, coiled-coil domain (CCD), DNA binding domain (DBD1), a linker domain (LD), a Src-homology 2 domain (SH2), a tyrosine phosphorylation site (pY) and a C-terminal transcriptional activation domain (TAD). The SH2 domain is critical for binding to a receptor (IL-4R or IL-13R) and consecutive activation of STAT6 through the phosphorylation of tyrosine residues with the intervention of Janus kinases (JAK), specifically Jak1 and Jak3. After the phosphorylation of receptors, STAT6 binds to them and is phosphorylated by Jak and TyK2 kinases on tyrosine residue Y641 located at the C-terminus of the SH2 domain, as indicated above. This entails the homodimerization of STAT6, which thereby translocates to the nucleus and efficiently binds to DNA sequences through the DBD, acting as a transcription factor (Figure 1) [75].

Even when at least 12 different *NAB2–STAT6* fusion variants have been described according to their breakpoints [76], the two most recurrent variants are: the *NAB2 exon4–STAT6 exon2* (N4S2), which is the most frequent, and the *NAB2 exon6–STAT6 exon16/17* (N6S16/17). N4S2 entails the fusion between NAB2 that lacks the CID domain and partially lacks the NCD2 domain, and the almost complete STAT6 part. This fusion correlates with a distinctive phenotype: primary tumors are derived mostly from the thoracic cavity, patients are older than in other breakpoint variants, tumors are significantly larger in diameter (median 10 cm) and they exhibit the appearance of a predominantly fibrotic and paucicellular tumor context. In contrast, the N6S16/17 fusion transcript contains almost all the NAB2 portion, except for exon 7 of CID, and a truncated STAT6 protein keeping TAD and part of the C-terminal of the SH2 domain. This N6S16/17 fusion is harbored more commonly by patients with pelvic, meningeal or extremity SFT, younger age than N4S2, smaller tumors (median 4.3 cm) and more tumor cellularity in comparison to N4S2 [76,77]. The prominent fibrosis seen in N4S2 could be related to the lack of CID (less repression on EGR1), and possibly to the presence of the almost entire STAT6 portion in the chimeric protein. Differential transcriptome analysis between the two most frequent fusion variants revealed that N4S2 exhibited a gene signature enriched for genes involved in DNA binding, gene transcription and nuclear localization, whereas the N6S16/17 signature was enriched for genes involved in tyrosine kinase signaling, cell proliferation and cytoplasmic localization [78]. Despite the fact that N4S2 correlates more frequently with less aggressive SFT, there is no convincing study demonstrating an unequivocal significant prognostic correlation among the fusion variants [69]. Larger studies analyzing homogeneous populations (i.e., completely resected localized tumors, or from metastatic spread) with enough follow-up are required. Additionally, the median tumor size of N4S2 was also greater, which would compensate the apparent better prognosis.

## 6. Biological Rationale for Antiangiogenic Therapy in SFT

The recognized involvement of CD34 in SFT has already been addressed above. This protein is expressed in endothelial progenitor and vascular cells, in addition to hematopoietic stem cells. In the late 1990s and early 2000s, pathologists reported the expression of vascular endothelial growth factor receptors (VEGFR) in tumor cells of SFT [79]. Most of them displayed positivity to VEGFR-1, while few were positive for VEGFR-2 (19%) or VEGFR-3 (31%) [80]. Recently, a more comprehensive analysis of receptor tyrosine kinase (RTK) expression was performed in 114 SFT patients. The majority of tumors expressed the following markers with an immunostaining intensity of at least 1+: VEGF expression in 82%, PDGFRα in 87%, PDGFRβ in 92%, PDGFα in 84% and PDGFβ in 96%. VEGFRs were not measured in this analysis. Interestingly, high expression (staining intensity 2+ or 3+) was significantly more frequently found for VEGF in hypercellular than hypocellular localized tumors, 32% vs. 8%, respectively, (*p* = 0.008) and the same occurred for PDGFβ, 41% vs. 13% (*p* = 0.008). Further, high expression of PDGFRα was significantly more frequent in metastatic than in localized SFT, 75% vs. 31% (*p* < 0.001). However, no significant prognostic correlation of these angiogenic factors was found for either the relapse-free survival or for overall survival (OS) [81]. The high expression of PDGFRα and PDGFRβ was not explained by mutational activation, since only two missense mutations of *PDGFRβ* were detected in a series of 88 SFT patients [58].

In 1999, it was established that *NAB2* was overexpressed, closely following the *EGR1* induction in vascular smooth cells in in vitro models, in response to phorbol 12-myristate 13-acetate (PMA) or to fibroblastic growth factor-2 (FGF-2), and more importantly, *NAB2* was able to inhibit *EGR1* in these cells. The same was detected in a vascular preclinical injury model. This was probably the first reported link between *NAB2, EGR1* and vascular endothelial homeostasis [82]. Along the same lines, a short time after it was observed in an in vitro model of angiogenesis that *NAB2* repressed *EGR1*-mediated growth factor production and secretion as VEGF, PDGFα/β, TGFβ, and HGF [83]. In endothelial cells, NAB2 was able to produce a strong inhibition of VEGF-induced expression of VEGFR [84]. Moreover, VEGFA and FGF2 mediate their biological roles through the transmembrane receptors VEGFR2 (for VEGFA) and FGFR1-4 (for FGF2), activating the MAPK pathway that ultimately upregulates EGR1 [85]. FGF2 also exerts as a key mediator which regulates EGR1 expression following the injury of endothelial cells [86]. In other words, some angiogenic factors are not just EGR1 targets, but are also able to stimulate EGR1 expression, highlighting the relevance of this signaling program in SFT, where EGR1 is derepressed to some extent. Another factor involved in angiogenesis and regulated by EGR1 is ISG-2 [87]. More recently, other key components of angiogenic signaling pathways such as EphrinB2-EphB4 and DLL4-Notch have been shown to be overexpressed in SFT, not only in endothelial cells but also in tumor cells [88].

On the other hand, STAT6 protein expression markedly increased immediately after arterial injury in an in vivo model. This overexpression was localized at the nuclear level and preceded the vascular smooth muscle cell proliferation. This overexpression declined from 7 days after the injury, coincident with the decline of vascular cell proliferation [89]. STAT6 protein expression also correlated with VEGF and the expression of microvessel density in nasal polyp tissues, this expression being significantly superior to turbinate mucosa tissues [90]. The identification of the DNA binding domain in STAT6B, analog of STAT6, mediated transcriptional regulation of *VEGF* was important for demonstrating a close link between this STAT6 analog and angiogenesis [91]. Taken as a whole, this information indicates that STAT6 overexpression could also induce the expression of *VEGF* in the context of SFT.

## 7. Biological Rationale for Not Using Doxorubicin in SFT (Except for DD-SFT)

Doxorubicin has been the first-line therapy in advanced soft-tissue sarcoma (STS) for more than 40 years, and despite several attempts and hundreds of randomized patients later, to date no doxorubicin-based combination has demonstrated a significant advantage in overall survival over doxorubicin alone [92,93,94,95]. The most recognized mechanisms of action for doxorubicin consist of its intercalation in the DNA that results in the disruption of DNA damage repair through topoisomerase II and the generation of free radicals with their ulterior cell membrane damage [96]. As already mentioned above, since SFT exhibits a phenotype of DD-SFT, significant genomic instability enters the scene with substantial cytogenetic losses and gains. Our group is investigating the role of proliferating cell nuclear antigen (PCNA), a critical component of the DNA damage tolerance (DDT) program in SFT genomic instability. This protein acts as scaffold recruiting components of DDT, in the evolving aggressiveness of the phenotype and complexity of the genotype. Interestingly, PCNA experiences post-translational modifications, such as ubiquitination, which triggers the exchange of DNA polymerase for the DNA damage-tolerant Y family. In addition to this, we are investigating the role of ISG15, a negative prognostic biomarker in advanced disease, in this ubiquitination as a crucial component for correcting genomic errors that could be accumulated by the permissive DDT system. PCNA immunostaining was positive in 50 and 80% of formerly named “typical” and “malignant” SFT, respectively, indicating a progressive presence towards aggressiveness [97]. It could be hypothesized that doxorubicin administration in the context of no dedifferentiated SFT could be detrimental, adding genomic instability through its direct genotoxic action or by doxorubicin-mediated ROS production. In fact, in an unpublished post hoc analysis of our data from a phase II study on pazopanib in SFT, low aggressive SFT patients that received doxorubicin-based chemotherapy as upfront therapy had a significantly shorter PFS when treated with pazopanib (9 vs. 24 months, *p* = 0.006) and a trend toward a worse overall survival (15 vs. 50 months, *p* = 0.093). Even when doxorubicin can induce a response irrespective of the cell cycle phase, it is more active in mitotic active cells. Furthermore, it is well known (see below) that the activity of doxorubicin in the context of SFT, where tumors barely exhibit mitotic activity, is negligible. Taken together, the previous information suggests that doxorubicin-based chemotherapy should be avoided in the case of SFT, not only due to the low efficacy but also because it could be detrimental, with the only exception being for DD-SFT.

## 8. Treatment in SFT

### 8.1. Surgery and Radiation Therapy

Surgical resection is the cornerstone of localized SFT treatment or in the oligometastatic setting. The sarcoma surgical approach has to be offered pursuing the goal of achieving wide resection. Since SFT is a ubiquitous tumor, technical aspects vary among different sites. In the case of thoracic SFT, it is most commonly attached to visceral pleura and usually pedunculated. Thus, pulmonary edge excision is the most frequently performed surgical resection. In SFT arising from parietal pleura or lungs, other resection types, such as isolated parietal pleural resection, chest wall resections or pulmonary wedge resections, are the most frequent procedures [98]. Video-assisted thoracic surgery, instead of thoracotomy, has been successfully performed especially in tumors smaller than 10 cm [99]. In the case of pedunculated tumors, an angiography to identify the pedicle which is rich in feeding vessels is recommended in order to perform a preoperative embolization or a thoracoscopy ligation of vessels. In sessile SFT, embolization is the most effective procedure in reducing intraoperative bleeding [100]. SFTs in the abdominal cavity are mainly located in the retroperitoneal space, with the involvement of the pelvic space being relatively frequent. The surgical approach is somewhat different to LPS since the growth pattern in SFT shows easily definable borders. Thus, adjacent organs can be spared as long as they are not encased, adherent or invaded [101]. Some authors report no prognostic difference between en bloc and piecemeal resections in pelvic SFT, although the follow-up of this series was just 43 months and they agree that the recurrence figures could be higher with a longer follow-up [102]. Concerning meningeal SFTs, almost all are attached to the dura mater with the most common location being along the tentorium cerebelli followed by the frontal convexity, cerebellopontine angle, ventricles, falx cerebri and posterior fossa [103]. Complete surgical resection entails clearly better prognosis than subtotal resection. However, microscopical margins are impossible to define as most tumors are fragmented during the resection [104]. This latter aspect along with the fact that meningeal SFTs more commonly exhibit hypercellularity and a higher mitotic count led to a worse prognosis compared to extrameningeal SFT. With respect to SFT of extremities, no special surgical approach, in comparison to other STSs, is required. The thigh is the most commonly involved site and tumors appear as a well-circumscribed primary and localized mass, usually in relation to fascial tissue. Adjuvant radiotherapy has usually been recommended in meningeal SFT, and even when no prospective study has been addressed, retrospective analysis suggests a clear trend toward significantly better local control if radiation therapy is added in the adjuvant setting. The 5-year local control in 89 patients was 60% vs. 90%, *p* = 0.052, favoring the postoperative radiotherapy (RT) group [105].

A large retrospective study including 549 SFT patients, 428 (78%) submitted to surgery and 121 (22%) submitted to surgery plus postoperative RT, revealed a significant benefit (*p* = 0.012) favoring RT in local control after propensity score matching. Nonetheless, no significant benefit was seen for OS [106]. Other smaller series have shown similar results [107,108]. Contrary to what was initially thought, SFT is sensitive to RT. Interestingly, a retrospective series of 40 patients treated with definitive RT (60 Gy) reported an overall response rate (ORR) of 67% with 5-year local control of 81.3% and 5-year OS of 87.5% [109]. Therefore, SFT cases at the limit of resectability, or those cases in which a marginal resection is foreseen, especially those cases with a high mitotic rate, could benefit from neoadjuvant RT. Further, the scheme of trabectedin plus low dose RT seems very active in several STS subtypes, [110] and could also be an alternative in cases requiring tumor shrinkage in order to facilitate limb sparing surgery, for instance. In any case, every treatment decision should be taken by a multidisciplinary team.

### 8.2. Chemotherapy

Chemotherapy has typically been used in the advanced or metastatic setting of SFT patients. However, very limited prospective evidence on the activity of standard cytotoxic drugs is available in SFT, and furthermore, no specific clinical trials addressing the value of chemotherapy have been reported on SFT. Several retrospective series have collected the activity of different cytotoxic drugs in the setting of advanced SFT (Table 2). Some of these series provided potential useful information regarding the different SFT subtypes included in the former 2013 STS classification of the WHO (typical, malignant and dedifferentiated subtypes) [111]. Preclinical studies with patient-derived xenografts (PDX) concluded that those cases diagnosed with DD-SFT are sensitive to chemotherapy [112]. Conversely, in terms of the value of cytotoxic drugs in those SFTs exhibiting less aggressive features, the formerly defined typical and malignant SFT, chemotherapy efficacy is much more controversial.

The main available evidence on chemotherapy concerns anthracycline-based regimens, with reported PFS in line with other STSs (3–5 months) and ORR ranging from 10.5–20% [113,114,115,116,117,118]. The value of the addition of ifosfamide is undetermined, although some data suggest longer PFS for combinations of doxorubicin plus ifosfamide [115]. In those series in which this information was available, DD-SFT patients obtained longer PFS results from anthracycline-based regimens when compared with malignant SFT patients [113] (Table 2). In the limited number of series reporting data on the activity of ifosfamide-based regimens, median PFS ranged from 2–3 months, and responses were reported in 2/19 patients (ORR 10%) in one of the series [113,118] (Table 3).

Dacarbazine (DTIC) and temozolomide have shown some positive results in SFT. DTIC has shown activity, alone or in combination with doxorubicin, in two DD-SFT PDX models, through the induction of DNA damage [112,119]. Interestingly, the preclinical activity of DTIC in these models was even superior to doxorubicin alone. Clinical evidence of activity has been retrospectively reported, both for temozolomide (in combination with bevacizumab) [123] and DTIC-based regimens. In detail, DTIC is the cytotoxic drug with the highest reported activity in terms of RECIST responses, ranging from 38% (in monotherapy) to 50% (in combination with doxorubicin) [112,119], and median PFS exceeded 6 months in the reported series, with a proportion of patients free of progression at 12 months. Given these promising results, the STRADA randomized phase II trial (NCT03023124) is currently evaluating the activity of doxorubicin plus DTIC in first line in patients with advanced SFT.

Trabectedin, a marine-derived alkaloid, is an approved drug for pretreated metastatic STS patients. This drug exhibits an interesting activity in translocation-related sarcomas (TRS), as is the case of SFT, apart from L-sarcomas [124]. Even if it has not been completely elucidated, trabectedin could act by modulating the transcription activity of the oncogenic fusion proteins in TRS [125,126]. Preclinical activity in SFT PDX models has been demonstrated [112], and several responses have been reported in retrospective series, with very variable PFS, ranging from 2.3 to 11.6 months [120,121,122]. The STRADA trial also includes a trabectedin arm, which will prospectively assess the activity of trabectedin.

Eribulin, a microtubule inhibitor, approved for second lines in liposarcoma patients [127], has also shown preclinical activity in in vivo SFT PDX models. Responses to eribulin have been reported in phase II trials on advanced STS: one patient with a malignant SFT included in the EORTC phase II trial [128] and one of the two patients with SFT included in the Japanese phase II trial [129]. Benefit from this drug has also been reported in four patients included in a retrospective Japanese series, with an interesting time to next line (as a surrogate of PFS) of 8 months [118]. In order to prospectively assess the efficacy of eribulin in SFT, a phase II trial is currently recruiting patients (NCT03023124).

Taken all together, chemotherapy could constitute a worthwhile option in those patients with DD-SFT. As mentioned earlier, concerns have emerged regarding converting SFT into tumors which are less sensitive to antiangiogenic agents after doxorubicin-based regimens. The results from the ongoing clinical trials will provide a very valuable piece of information, especially if post-protocol therapies and full-length overall survival are collected.

### 8.3. Antiangiogenics

The data published on the activity of antiangiogenics in SFT are summarized in Table 3.

The combination of temozolomide and bevacizumab, a recombinant monoclonal antibody that targets VEGF, was one of the first pieces of evidence for the antiangiogenic activity in SFT [123]. This retrospective experience compiled a single-center short series of 14 patients, reporting 11 partial responses (79%), 2 stable disease (14%) and 1 progressive disease (7%) according to Choi criteria. There were 2 partial responses and 12 stable disease following RECIST criteria. The median of PFS was 9.7 months and 10.8 according to Choi and RECIST, respectively, whereas the median OS was 24.3 months. This series could carry a bias related to a favorable biological behavior (there were three low aggressive SFTs and six with unknown aggressiveness) and widely ranged radiological assessments between 8 and 12 weeks that could have some influence on the PFS (Table 4).

Sunitinib, a multi-tyrosine kinase inhibitor affecting VEGFR1-3, PDGFRβ, Fms-like tyrosine kinase 3 (Flt3), KIT and rearranged during transfection (RET), was reported in a retrospective series of 35 unfavorable SFT patients from a single institution [130]. Thirty-one patients (20 formerly labeled as malignant, and 11 DD-SFT) were evaluable for RECIST and 29 (19 malignant, 10 DD-SFT) were evaluable for Choi criteria. The responses according to RECIST and Choi were as follows: PR 6.5%, SD 54%, PD 39.5% and PR 48%, SD 17%, PD 34%, respectively. The median PFS for Choi and RECIST was 7 months (95% CI 5–10.3) and 6 months (95% CI 4.03–8.01), respectively, with a median OS of 16 months (95% CI 12.07–25.9). In this experience, the authors found that DD-SFT cases (48% of the series) had less sensitivity than those labeled as malignant SFT. Even when the intervals of radiological assessment seemed to be as relaxed as in previous series, every 3 months from the third month on, the patients of this study exhibited bad prognostic features (high grade SFT, with a substantial percentage of DD-SFT and 25 patients previously treated with chemotherapy). Thus, this study offers a remarkable outcome for sunitinib in SFT. Previously, other authors had noticed the long-lasting stabilizations with sunitinib [132,133,134]. The combination of antiangiogenic (sunitinib) plus anti PD-1 (nivolumab) has been explored in several sarcomas including SFT, in the IMMUNOSARC-1 trial [135]. Seven SFT patients were enrolled obtaining an mPFS of 7.5 months; apparently no big differences were obtained with respect to the use of antiangiogenics alone.

Sorafenib, a multi-kinase inhibitor that targets VEGFR 1–3, PDGFRβ, FGFR and RAF among others, was reported as an active agent in five advanced and progressive patients with SFT [136]. The median PFS was 5.9 months, with two long-lasting SD, while the median OS was 19.7 months.

Pazopanib, a multi-targeted receptor tyrosine kinase inhibitor against VEGFR 1–3, PDGFR and KIT, with a modest activity against fibroblast growth factor receptor (FGFR) 1–3, was first reported as active in SFT in a prospective short collection of 13 patients from a single institution [131]. Radiologic assessment for 11 evaluable patients revealed one PR (9%), eight SD (73%), two PD (18%) and five PR (46%), four SD (36%), two PD (18%) for RECIST and CHOI criteria, respectively. The median PFS was 4.7 months and the median OS was 13.3 months. No information about pathologic aggressiveness was reported that could explain the worse outcome in comparison with retrospective series treated with sunitinib.

**Figure 3:**
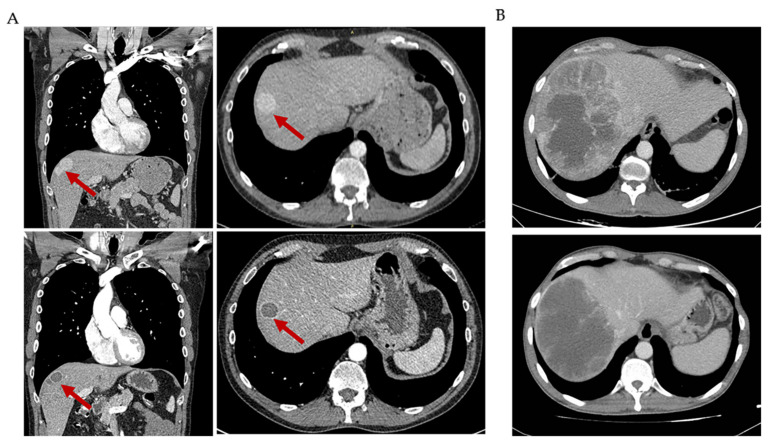
Choi responses to antiangiogenic agents in solitary fibrous tumor (SFT). (**A**) CT scan of an SFT patient showing a characteristic Choi partial response to pazopanib treatment in one hepatic lesion: decrease in density of 56%. (**B**) CT scan of an SFT patient showing a characteristic Choi partial response to pazopanib treatment in one hepatic lesion: decrease in density of 33%.

Pazopanib was also tested in the first clinical trial (GEIS-32) conducted in two different SFT cohorts, those formerly called typical and malignant SFT. In the aggressive SFT series (malignant and DD-SFT), 36 patients were enrolled in 16 centers across Italy, France and Spain. From 35 evaluable patients and following the central radiology assessment, there were 18 (51%) PR, 9 (26%) SD and 8 (23%) PD according to Choi criteria, whereas there were 2 (6%) PR, 21 (60%) SD and 12 (34%) PD according to RECIST criteria [65]. An example of Choi response is given in Figure 3. The median decrease in tumor density was 28%. The median PFS was 5.57 months by both Choi and RECIST response and the median OS was not reached (median follow-up of 27 months), though the 2-year OS rate was 73% (95% CI 58–88%). The protocol was amended to avoid the enrollment of DD-SFT, after confirming two fast progressions in the first two DD-SFT patients. Previous systemic treatment was received by 33% of accrued patients. Post-protocol therapies were advised with rotating antiangiogenics if at least an SD was achieved as the best response. The outcome of this trial, in addition to offering a benchmark of activity in SFT, compares favorably to the clinical results obtained with chemotherapy.

On the other hand, in the cohort of low aggressive (formerly typical) SFT, 34 patients were enrolled and received pazopanib. From them, 31 patients were evaluable for efficacy endpoints. According to Choi central evaluation, there were 18 (58%) PR and 12 (39%) SD and 1 (3%) PD, and according to RECIST there were 2 (6%) PR, and 29 (94%) SD [21]. Of note, the median PFS following Choi and RECIST and central radiological assessment was 9.8 months (95% CI, 7.2–12.3) and 11.2 months (95% CI, 9.1–13.4), respectively. Interestingly, the longer PFS verified in this cohort compared to the more aggressive (formerly malignant) cohort could be explained by the more indolent biology that would still be maintained even when metastatic spread has appeared. The suggested correlation between NAB2ex6–STAT6ex16/17 with vascular markers [78] did not explain this difference for pazopanib PFS detected in both cohorts.

Axitinib, another oral multi-tyrosine kinase inhibitor that actively targets VEGFR 1–3, PDGFRβ and KIT, was prospectively tested in a single-center trial recruiting 17 evaluable patients. The response assessment according to Choi showed seven PR (41.2%), six SD (35.3%) and four PD (23.5%). Interestingly, partial responses were seen in four out of nine (44.4%) among those previously treated with antiangiogenics, which indicates the value of rotating antiangiogenics at the time of progression in SFT, as indicated. Of note, none of the DD-SFT patients responded to axitinib in line with the observation with pazopanib.

In conclusion, in non-dedifferentiated SFT, antiangiogenic agents are more active, by indirect comparison, than chemotherapy. Pazopanib is the recommendation as first line, based on its favorable toxicity profile and the efficacy derived from a phase II trial. At the time of progression, rotating antiangiogenics is a reasonable approach since new Choi responses have been observed with this strategy. Further, the survival length seems superior compared with the historical use of chemotherapy. Other antiangiogenics, such as sunitinib or axitinib, have demonstrated activity in SFT and could be used sequentially. The comparative inhibitory effect of some of these tyrosine kinase inhibitors on RTK is shown in Table 5. Regarding the use of doxorubicin-based regimens in non-dedifferentiated SFT, we have found in the prospective data collected in the GEIS-32 trial that it was detrimental to the antiangiogenic therapy. Our recommendation is to use them in the context of antiangiogenic refractory SFT, especially dacarbazine-based chemotherapy. Trabectedin could also be an option to consider for the treatment of progressing SFT.

## 9. Future Perspectives

As new insights on critical signaling mechanisms are being reported, new treatment opportunities can be devised in SFT. Targeting EGR1 seems a reasonable future approach in SFT, since it is a critical axis in the signaling of the fusion transcript *NAB2–STAT6*. Although it was considered an interesting candidate for prostate cancer years ago [145], no further evolution has been addressed in this line. Following our finding that RNA expression of preferentially expressed antigen in melanoma (*PRAME*) was related to worse prognosis in SFT [65], along with the finding of PRAME positive protein expression in 165 out of 180 SFTs, with high expression seen in 58% [146], this makes it an attractive target for TCR-T cell therapies. Therapy with anti PD-1 drugs has not yet been explored in the context of DD-SFT. In theory, in a similar way to DD chondrosarcoma for which this immunomodulation is active, which could be related to high tumor mutational burden [147] in the context of a complex karyotype due to genomic instability, this approach could also be effective in DD-SFT for the same reason. Another attractive therapeutic approach is the combination of trabectedin plus low-dose radiation therapy [110], especially knowing that SFT is radiosensitive, and that the combination is safe and remarkably active in the metastatic setting. Targeting the protein transcript could be possible, as has recently been reported in the *EWSR1-FLI1*-translocated Ewing sarcoma [148]. That could be mimicked to some extent in the two most frequent fusion transcript breakpoints for *NAB2–STAT6*. Other EGR1-related targets, such as IGF2 or FGFR for instance, could also be alternative targets to explore in SFT. Finally, other molecules, such as CD99 expressed in an early stage in SFT, could also be a therapeutic target in SFT [149].

## Figures and Tables

**Table 1:** Univariate and multivariate analysis of series (analyzed localized resected cases).

Author	N Total/Selected	External Validation	Subtypes	RFS/MFS/DFS		Overall Survival	
				Univariate	Multivariate	Univariate	Multivariate
Salas et al. [9]	214/162	Yes	Extrameningeal	Age, Mitosis, Necrosis	Mitosis	Age, Mitosis	Age
Pasquali et al. [22]	269/243	No	Extrameningeal and Extrapleural	Mitosis, Necrosis, Hypercellularity, Pleomorphism, Positive margins	Mitosis, Hypercellularity, Pleomorphism	Mitosis, Necrosis, Hypercellularity, Pleomorphism, Age, deep-seated	Pleomorphism, Hypercellularity
Demicco et al. [6]	110	Yes	Extrameningeal	Age, Mitosis, Size, Necrosis	Mitosis, Size	Age, Size, Mitosis	NA
Gholami et al. [25]	219	No	Extrameningeal	Size	NA	T location, Size	T location, Size
Tapias et al. [23]	59	No	Pleural	Size, Hypercellularity, Necrosis, Mitosis, Pleural effusion, Parietal (vs. visceral)	Size	NA	NA
Reisenauer et al. [27]	147	NA	Pleuro-Pulmonary	NA	NA	Age, Sex, Pattern *, Pleomorphism, Necrosis, Mitosis, Size	NA
Georgiesh et al. [24]	100	No	Extrameningeal	Sex, Necrosis, Mitosis, Pleomorphism	Mitosis, Necrosis, Sex	Age, Mitosis, Necrosis, Pleomorphism	Age, Mitosis, Necrosis,

RFS: relapse free survival; DFS: disease free survival; MFS: metastasis free survival; (*) nonpedunculated vs. pedunculated pattern. NA: Not applicable.

**Table 2:** Immunohistochemical markers commonly expressed in solitary fibrous tumors.

Marker	Normal Tissues/Precursors	Mesenchymal Benign Entities	Mesenchymal Malignant Tumors
CD 34	-Early hematopoietic stem cells	- Giant cell fibroblastoma - Lipoma	-SFT *
	-Mesenchymal stem cells		-Kaposi sarcoma
	-Small-vessels endothelial cells		-Low-grade myofibroblastic sarcoma
	-Embryonic fibroblasts		-Inflammatory myofibroblastic tumors
	-Endoneurial cells, dermal dendritic interstitial fibroblastic cells		-DFSP
	-Adipocitic cells		-GIST
bcl 2	-Stem cells -Endocrine tissue -Long-lived cells	-Schwannoma -Spindle cell lipoma -Dendritic fibromyxolipoma -Neurofibromas (focal)	-SFT * -Fibrosarcoma -Low-grade myxofibrosarcoma -Dedifferentiated liposarcoma -Synovial sarcoma -DFSP -GIST -Kaposi sarcoma -MPNST
CD99	-Bone marrow cells -Leukocytes	-Fibroma -Giant cell angiofibroma	-SFT * -Ewing sarcoma -Synovial sarcoma -Rhabdomyosarcoma -Osteosarcoma
STAT 6			-SFT * -Unclassified sarcomas of spindle cell or epithelioid morphology (12%) -Desmoid tumors (8%) -Neurofibromas (5%) -Clear cell sarcoma (5%) -Well-diff./dedifferentiated liposarcoma

* Expression can be lost in dedifferentiated solitary fibrous tumors (SFT); DFSP: Dermatofibrosarcoma protuberans; GIST: Gastrointestinal stromal tumors; MPSNT: Malignant peripheral stealth nerve tumors.

**Table 3:** Data on chemotherapy for advanced solitary fibrous tumor.

Series	Type of Study	*n*	Classification of SFT	Drug/Regimen	Responses	mPFS (Months)	mOS (Months)
Stacchiotti et al. 2013 [113]	Retrospective	31	Typical: 1 MSFT: 17 DSFT: 12 NA: 1	Anthracycline-based (30 pts): 8 monotherapy 23 Anthracycline + Ifosfamide High-dose Ifosfamide (19 pts)	PR: 6 (20%); SD: 8 (27%); PD: 16 (53%) PR: 2 (10%) SD: 5 (26%) PD: 12 (63%)	3.5 (MSFT) 5 (DSFT) 3	11.5 NR
Levard et al. 2013 [115]	Retrospective	23	NR	Anthracycline-based (19 pts): 9 monotherapy 9 Doxo-Ifo/palifosfamide 1 Pegylated liposomal doxorubicin	PR: 2/19 (10.5%)	4 (Doxo) 6.7 (combination)	NR
Constantinidou et al. 2012 [114]	Retrospective	24	NR	Anthracycline-based (17 pts): 14 monotherapy 3 Anthracycline + IFO Ifosfamide (4)	PR: 1/17 (6%) PR: 0; SD: 3/4 (75%); PD: 1 (25%)	4.2 NR	14.6 NR
Schöffski et al. 2020 [116]	Retrospective	94 (26 treated)	NR	Doxorubicin (15 pts)	PR: 2 (13%) SD: 4 (26%) PD: 7 (47%) NE: 2 (13%)	4.8	NR
Stacchiotti et al. 2017 [112]	Retrospective	12	MSFT: 7 DSFT: 5	Doxorubicin + Dacarbazine	PR: 6 (50%) SD: 1 (8.3%) PD: 5 (41.7%)	6 (MSFT) 10 (DSFT)	19
Outani et al. 2020 [118]	Retrospective	60 (31 treated)	Typical: 7 (12%) MSFT: 35 (58%) NA: 18 (30%)	Anthracycline-based (11 pts) Gemcitabine–docetaxel (10 pts) Ifosfamide-based (7 pts) Trabectedin (6 pts) Eribulin (4 pts)	NR	3 * 8 * 2 * 3.5 * 8 *	NR
Park et al. 2013 [117]	Retrospective	21	NR	Anthracycline-based (15 pts) 1 monotherapy 14 Doxorubicin combos Gemcitabine-based (5 pts) Paclitaxel (3 pts)	PR: 0; SD: 14/15 (93%); PD: 1/15 (7%) SD: 2/5 (40%); PD: 3/5 (40%) SD: 4/5 (80%); PD: 1/5 (20%)	4.6 **	10.3 ***
Stacchiotti et al. 2013 [119]	Retrospective	8	MSFT: 3 DSFT: 5	Dacarbazine	PR: 3 (38%) SD: 4 (50%) PD: 1 (12%)	7	NR
Chaigneau et al. 2011 [120]	Case report	1	MSFT	Trabectedin	PR	8	NR
Khalifa et al. 2015 [121]	Retrospective	11	Typical: 1 MSFT: 10	Trabectedin	PR: 1 (9.1%) SD: 8 (72.7%)	11.6	22.3
Kobayashi et al. 2020 [122]	Retrospective	6	NR	Trabectedin	PR: 1 (16.7%)	2.3	NR

SFT: Solitary Fibrous Tumor; MSFT: Malignant SFT; DSFT: Dedifferentiated SFT; PR: Partial response; SD: Stable disease; PD: Progressive disease; NR: Not reported; Pts: patients; mPFS: median progression-free survival; mOS: median overall survival: NA: not assessable. * Expressed as time to next treatment (TNT); ** Results for all drugs pooled; *** OS from diagnosis for all the series.

**Table 4:** Activity of antiangiogenics in solitary fibrous tumor.

Author	N	Design	Scheme	mPFS (Months)	6mPFSR	SFT Subtype	Response	
							RECIST	Choi
Stacchiotti et al. 2013 [113]	31	Retrospective	Anthracycline-based	3.5 (M SFT) 4.0 (DD-SFT)	20%	17 M SFT; 12 DD-SFT	PR 20% SD 27%	UNK
Park et al. 2013 [117]	25	Retrospective	Anthracycline-based and others	4.6	28%	UNK	SD 80%	UNK
Park et al. 2011 [123]	14	Retrospective	Temozolomide and Bevacizumab	9.7	78.6%	3 T SFT; 5 M SFT; 6 UNK	PR 14% SD 86%	PR 79% SD 14% PD 7%
Stacchiotti et al. 2012 [130]	35	Retrospective	Sunitinib	6	45%	22 M SFT; 13 DD-SFT	PR 6% SD 54% PD 40%	PR 48% SD 17% PD 34%
Maruzzo et al. 2015 [131]	13	Prospective collection	Pazopanib	4.7	44.9%	UNK	PR 9% SD 73% PD 18%	PR 46% SD 36% PD 18%

**Table 5:** Comparative inhibitory effect of some tyrosine kinase inhibitors.

IC_50_ (nM) in Cell-Free Kinase Assay										
Inhibitor	VEGFR1	VEGFR2	VEGFR3	PDGFRα	PDGFRβ	FGFR1	FGFR2	FGFR3	FGFR4	C-Met
Anlotinib [137]	26.9	0.2	0.7	ND	115	ND	ND	ND	ND	>1000
Axitinib [138]	ND	0.2	ND	5	1.6	ND	ND	ND	ND	ND
Cabozantinib [139]	ND	0.035	ND	ND	234	>1000	ND	ND	ND	1.3
Lenvatinib [140]	22	4	5.2	51	39	46	ND	ND	ND	ND
Pazopanib [141]	10	30	47	71	84	140	ND	130	800 *	ND
Regorafenib [142]	13	4.2	46	ND	22	202	ND	ND	ND	ND
Sorafenib [143]	ND	90	ND	ND	57	580	ND	ND	ND	>1000
Sunitinib [144]	21	34	3	ND	75	437	ND	ND	ND	ND

ND: not determined; * estimated.

## References

[1] Kinslow C.J., Wang T.J.C. (2020). Incidence of extrameningeal solitary fibrous tumors. Cancer.

[2] Kinslow C.J., Bruce S.S., Rae A.I., Sheth S.A., McKhann G.M., Sisti M.B., Bruce J.N., Sonabend A.M., Wang T.J.C. (2018). Solitary-fibrous tumor/hemangiopericytoma of the central nervous system: A population-based study. J. Neuro-Oncol..

[3] Fletcher C.D. (2014). The evolving classification of soft tissue tumours—An update based on the new 2013 WHO classification. Histopathology.

[4] Kallen M.E., Hornick J.L. (2021). The 2020 WHO Classification: What’s New in Soft Tissue Tumor Pathology?. Am. J. Surg. Pathol..

[5] O’Neill A.C., Tirumani S.H., Do W.S., Keraliya A.R., Hornick J.L., Shinagare A.B., Ramaiya N.H. (2017). Metastatic Patterns of Solitary Fibrous Tumors: A Single-Institution Experience. AJR. Am. J. Roentgenol..

[6] Demicco E.G., Park M.S., Araujo D.M., Fox P.S., Bassett R.L., Pollock R.E., Lazar A.J., Wang W.L. (2012). Solitary fibrous tumor: A clinicopathological study of 110 cases and proposed risk assessment model. Mod. Pathol..

[7] Baldi G.G., Stacchiotti S., Mauro V., Dei Tos A.P., Gronchi A., Pastorino U., Duranti L., Provenzano S., Marrari A., Libertini M. (2013). Solitary fibrous tumor of all sites: Outcome of late recurrences in 14 patients. Clin. Sarcoma Res..

[8] Fritchie K., Jensch K., Moskalev E.A., Caron A., Jenkins S., Link M., Brown P.D., Rodriguez F.J., Guajardo A., Brat D. (2019). The impact of histopathology and NAB2-STAT6 fusion subtype in classification and grading of meningeal solitary fibrous tumor/hemangiopericytoma. Acta Neuropathol..

[9] Salas S., Resseguier N., Blay J.Y., Le Cesne A., Italiano A., Chevreau C., Rosset P., Isambert N., Soulie P., Cupissol D. (2017). Prediction of local and metastatic recurrence in solitary fibrous tumor: Construction of a risk calculator in a multicenter cohort from the French Sarcoma Group (FSG) database. Ann. Oncol..

[10] Callemeyn J., Van Haecke P., Peetermans W.E., Blockmans D. (2016). Clubbing and hypertrophic osteoarthropathy: Insights in diagnosis, pathophysiology, and clinical significance. Acta Clin. Belg..

[11] Dickinson C.J., Martin J.F. (1987). Megakaryocytes and platelet clumps as the cause of finger clubbing. Lancet.

[12] Atkinson S., Fox S.B. (2004). Vascular endothelial growth factor (VEGF)-A and platelet-derived growth factor (PDGF) play a central role in the pathogenesis of digital clubbing. J. Pathol..

[13] Olan F., Portela M., Navarro C., Gaxiola M., Silveira L.H., Ruiz V., Martinez-Lavin M. (2004). Circulating vascular endothelial growth factor concentrations in a case of pulmonary hypertrophic osteoarthropathy. Correlation with disease activity. J. Rheumatol..

[14] Le Roith D. (1999). Tumor-induced hypoglycemia. N. Engl. J. Med..

[15] Wignall O.J., Moskovic E.C., Thway K., Thomas J.M. (2010). Solitary fibrous tumors of the soft tissues: Review of the imaging and clinical features with histopathologic correlation. Am. J. Roentgenol..

[16] Helage S., Revel M.P., Chabi M.L., Audureau E., Ferretti G., Laurent F., Alifano M., Mansuet-Lupo A., Buy J.N., Vadrot D. (2016). Solitary fibrous tumor of the pleura: Can computed tomography features help predict malignancy? A series of 56 patients with histopathological correlates. Diagn. Interv. Imaging.

[17] Rosado-de-Christenson M.L., Abbott G.F., McAdams H.P., Franks T.J., Galvin J.R. (2003). From the archives of the AFIP: Localized fibrous tumor of the pleura. Radiographics.

[18] Ginat D.T., Bokhari A., Bhatt S., Dogra V. (2011). Imaging features of solitary fibrous tumors. Am. J. Roentgenol..

[19] Tazeler Z., Tan G., Aslan A., Tan S. (2016). The utility of 18F-FDG PET/CT in solitary fibrous tumors of the pleura. Rev. Esp. Med. Nucl. Imagen Mol..

[20] WHO Classification of Tumours Editorial Board (2020). Soft Tissue and Bone Tumours. WHO Classification of Tumours Series.

[21] Martin-Broto J., Cruz J., Penel N., Le Cesne A., Hindi N., Luna P., Moura D.S., Bernabeu D., de Alava E., Lopez-Guerrero J.A. (2020). Pazopanib for treatment of typical solitary fibrous tumours: A multicentre, single-arm, phase 2 trial. Lancet Oncol..

[22] Pasquali S., Gronchi A., Strauss D., Bonvalot S., Jeys L., Stacchiotti S., Hayes A., Honore C., Collini P., Renne S.L. (2016). Resectable extra-pleural and extra-meningeal solitary fibrous tumours: A multi-centre prognostic study. Eur. J. Surg. Oncol..

[23] Tapias L.F., Mino-Kenudson M., Lee H., Wright C., Gaissert H.A., Wain J.C., Mathisen D.J., Lanuti M. (2013). Risk factor analysis for the recurrence of resected solitary fibrous tumours of the pleura: A 33-year experience and proposal for a scoring system. Eur. J. Cardio-Thorac. Surg..

[24] Georgiesh T., Boye K., Bjerkehagen B. (2020). A novel risk score to predict early and late recurrence in solitary fibrous tumour. Histopathology.

[25] Gholami S., Cassidy M.R., Kirane A., Kuk D., Zanchelli B., Antonescu C.R., Singer S., Brennan M. (2017). Size and Location are the Most Important Risk Factors for Malignant Behavior in Resected Solitary Fibrous Tumors. Ann. Surg. Oncol..

[26] Demicco E.G., Wagner M.J., Maki R.G., Gupta V., Iofin I., Lazar A.J., Wang W.L. (2017). Risk assessment in solitary fibrous tumors: Validation and refinement of a risk stratification model. Mod. Pathol..

[27] Reisenauer J.S., Mneimneh W., Jenkins S., Mansfield A.S., Aubry M.C., Fritchie K.J., Allen M.S., Blackmon S.H., Cassivi S.D., Nichols F.C. (2018). Comparison of Risk Stratification Models to Predict Recurrence and Survival in Pleuropulmonary Solitary Fibrous Tumor. J. Thorac. Oncol..

[28] Lee J.C., Fletcher C.D. (2011). Malignant fat-forming solitary fibrous tumor (so-called “lipomatous hemangiopericytoma”): Clinicopathologic analysis of 14 cases. Am. J. Surg. Pathol..

[29] Dong S.S., Wang N., Yang C.P., Zhang G.C., Liang W.H., Zhao J., Qi Y. (2020). Giant Cell-Rich Solitary Fibrous Tumor in the Nasopharynx: Case Report and Literature Review. OncoTargets Ther..

[30] Thway K., Hayes A., Ieremia E., Fisher C. (2013). Heterologous osteosarcomatous and rhabdomyosarcomatous elements in dedifferentiated solitary fibrous tumor: Further support for the concept of dedifferentiation in solitary fibrous tumor. Ann. Diagn. Pathol..

[31] Doyle L.A., Vivero M., Fletcher C.D., Mertens F., Hornick J.L. (2014). Nuclear expression of STAT6 distinguishes solitary fibrous tumor from histologic mimics. Mod. Pathol..

[32] Dagrada G.P., Spagnuolo R.D., Mauro V., Tamborini E., Cesana L., Gronchi A., Stacchiotti S., Pierotti M.A., Negri T., Pilotti S. (2015). Solitary fibrous tumors: Loss of chimeric protein expression and genomic instability mark dedifferentiation. Mod. Pathol..

[33] Barron L., Wynn T.A. (2011). Fibrosis is regulated by Th2 and Th17 responses and by dynamic interactions between fibroblasts and macrophages. Am. J. Physiol. Gastrointest. Liver Physiol..

[34] Ingram J.L., Antao-Menezes A., Mangum J.B., Lyght O., Lee P.J., Elias J.A., Bonner J.C. (2006). Opposing actions of Stat1 and Stat6 on IL-13-induced up-regulation of early growth response-1 and platelet-derived growth factor ligands in pulmonary fibroblasts. J. Immunol..

[35] Demicco E.G., Harms P.W., Patel R.M., Smith S.C., Ingram D., Torres K., Carskadon S.L., Camelo-Piragua S., McHugh J.B., Siddiqui J. (2015). Extensive survey of STAT6 expression in a large series of mesenchymal tumors. Am. J. Clin. Pathol..

[36] Doyle L.A., Tao D., Marino-Enriquez A. (2014). STAT6 is amplified in a subset of dedifferentiated liposarcoma. Mod. Pathol..

[37] Ouladan S., Trautmann M., Orouji E., Hartmann W., Huss S., Buttner R., Wardelmann E. (2015). Differential diagnosis of solitary fibrous tumors: A study of 454 soft tissue tumors indicating the diagnostic value of nuclear STAT6 relocation and ALDH1 expression combined with in situ proximity ligation assay. Int. J. Oncol..

[38] Yokoi T., Tsuzuki T., Yatabe Y., Suzuki M., Kurumaya H., Koshikawa T., Kuhara H., Kuroda M., Nakamura N., Nakatani Y. (1998). Solitary fibrous tumour: Significance of p53 and CD34 immunoreactivity in its malignant transformation. Histopathology.

[39] Waller E.K., Olweus J., Lund-Johansen F., Huang S., Nguyen M., Guo G.R., Terstappen L. (1995). The “common stem cell” hypothesis reevaluated: Human fetal bone marrow contains separate populations of hematopoietic and stromal progenitors. Blood.

[40] Quirici N., Soligo D., Bossolasco P., Servida F., Lumini C., Deliliers G.L. (2002). Isolation of bone marrow mesenchymal stem cells by anti-nerve growth factor receptor antibodies. Exp. Hematol..

[41] De Francesco F., Tirino V., Desiderio V., Ferraro G., D’Andrea F., Giuliano M., Libondi G., Pirozzi G., De Rosa A., Papaccio G. (2009). Human CD34/CD90 ASCs are capable of growing as sphere clusters, producing high levels of VEGF and forming capillaries. PLoS ONE.

[42] Hasegawa T., Matsuno Y., Shimoda T., Hirohashi S., Hirose T., Sano T. (1998). Frequent expression of bcl-2 protein in solitary fibrous tumors. Jpn. J. Clin. Oncol..

[43] Takizawa I., Saito T., Kitamura Y., Arai K., Kawaguchi M., Takahashi K., Hara N. (2008). Primary solitary fibrous tumor (SFT) in the retroperitoneum. Urol. Oncol..

[44] Suster S., Fisher C., Moran C.A. (1998). Expression of bcl-2 oncoprotein in benign and malignant spindle cell tumors of soft tissue, skin, serosal surfaces, and gastrointestinal tract. Am. J. Surg. Pathol..

[45] Baer R. (1994). Bcl-2 breathes life into embryogenesis. Am. J. Pathol..

[46] Aronica M.A., Goenka S., Boothby M. (2000). IL-4-dependent induction of BCL-2 and BCL-X(L)IN activated T lymphocytes through a STAT6- and pi 3-kinase-independent pathway. Cytokine.

[47] Hirakawa N., Naka T., Yamamoto I., Fukuda T., Tsuneyoshi M. (1996). Overexpression of bcl-2 protein in synovial sarcoma: A comparative study of other soft tissue spindle cell sarcomas and an additional analysis by fluorescence in situ hybridization. Hum. Pathol..

[48] Manara M.C., Pasello M., Scotlandi K. (2018). CD99: A Cell Surface Protein with an Oncojanus Role in Tumors. Genes.

[49] Maitra A., Hansel D.E., Argani P., Ashfaq R., Rahman A., Naji A., Deng S., Geradts J., Hawthorne L., House M.G. (2003). Global expression analysis of well-differentiated pancreatic endocrine neoplasms using oligonucleotide microarrays. Clin. Cancer Res..

[50] Manara M.C., Bernard G., Lollini P.L., Nanni P., Zuntini M., Landuzzi L., Benini S., Lattanzi G., Sciandra M., Serra M. (2006). CD99 acts as an oncosuppressor in osteosarcoma. Mol. Biol. Cell.

[51] Scotlandi K., Zuntini M., Manara M.C., Sciandra M., Rocchi A., Benini S., Nicoletti G., Bernard G., Nanni P., Lollini P.L. (2007). CD99 isoforms dictate opposite functions in tumour malignancy and metastases by activating or repressing c-Src kinase activity. Oncogene.

[52] Bremond A., Meynet O., Mahiddine K., Coito S., Tichet M., Scotlandi K., Breittmayer J.P., Gounon P., Gleeson P.A., Bernard A. (2009). Regulation of HLA class I surface expression requires CD99 and p230/golgin-245 interaction. Blood.

[53] Olson N.J., Linos K. (2018). Dedifferentiated Solitary Fibrous Tumor: A Concise Review. Arch. Pathol. Lab. Med..

[54] Han Y., Zhang Q., Yu X., Han X., Wang H., Xu Y., Qiu X., Jin F. (2015). Immunohistochemical detection of STAT6, CD34, CD99 and BCL-2 for diagnosing solitary fibrous tumors/hemangiopericytomas. Int. J. Clin. Exp. Pathol..

[55] Fletcher C.D.M., Bridge J.A., Lee J.C., Fletcher C.D.M., Bridge J.A., Hogendoom P.C.W., Mertens F. (2013). Extrapleural solitary fibrous tumor. WHO Classification of Tumors of Soft Tissue and Bone.

[56] Yang J.W., Song D.H., Jang I.S., Ko G.H. (2014). Dedifferentiated solitary fibrous tumor of thoracic cavity. Korean J. Pathol..

[57] Schneider N., Hallin M., Thway K. (2017). STAT6 Loss in Dedifferentiated Solitary Fibrous Tumor. Int. J. Surg. Pathol..

[58] Schirosi L., Lantuejoul S., Cavazza A., Murer B., Yves Brichon P., Migaldi M., Sartori G., Sgambato A., Rossi G. (2008). Pleuro-pulmonary solitary fibrous tumors: A clinicopathologic, immunohistochemical, and molecular study of 88 cases confirming the prognostic value of de Perrot staging system and p53 expression, and evaluating the role of c-kit, BRAF, PDGFRs (alpha/beta), c-met, and EGFR. Am. J. Surg. Pathol..

[59] Kurisaki-Arakawa A., Akaike K., Hara K., Arakawa A., Takahashi M., Mitani K., Yao T., Saito T. (2014). A case of dedifferentiated solitary fibrous tumor in the pelvis with TP53 mutation. Virchows Arch..

[60] Akaike K., Kurisaki-Arakawa A., Hara K., Suehara Y., Takagi T., Mitani K., Kaneko K., Yao T., Saito T. (2015). Distinct clinicopathological features of NAB2-STAT6 fusion gene variants in solitary fibrous tumor with emphasis on the acquisition of highly malignant potential. Hum. Pathol..

[61] Hu F., Xue M., Li Y., Jia Y.J., Zheng Z.J., Yang Y.L., Guan M.P., Sun L., Xue Y.M. (2018). Early Growth Response 1 (Egr1) Is a Transcriptional Activator of NOX4 in Oxidative Stress of Diabetic Kidney Disease. J. Diabetes Res..

[62] Arakawa Y., Miyake H., Horiguchi H., Inokuchi T., Hino N., Ogasawara T., Kuroda T., Yamasaki S. (2018). Overexpression of IGF2 and IGF2 receptor in malignant solitary fibrous tumor with hypoglycemia: A case report. Surg. Case Rep..

[63] Yamada Y., Kohashi K., Kinoshita I., Yamamoto H., Iwasaki T., Yoshimoto M., Ishihara S., Toda Y., Itou Y., Koga Y. (2019). Clinicopathological review of solitary fibrous tumors: Dedifferentiation is a major cause of patient death. Virchows Arch..

[64] England D.M., Hochholzer L., McCarthy M.J. (1989). Localized benign and malignant fibrous tumors of the pleura. A clinicopathologic review of 223 cases. Am. J. Surg. Pathol..

[65] Martin-Broto J., Stacchiotti S., Lopez-Pousa A., Redondo A., Bernabeu D., de Alava E., Casali P.G., Italiano A., Gutierrez A., Moura D.S. (2019). Pazopanib for treatment of advanced malignant and dedifferentiated solitary fibrous tumour: A multicentre, single-arm, phase 2 trial. Lancet Oncol..

[66] Chen R.H., Du Y., Han P., Wang H.B., Liang F.Y., Feng G.K., Zhou A.J., Cai M.Y., Zhong Q., Zeng M.S. (2016). ISG15 predicts poor prognosis and promotes cancer stem cell phenotype in nasopharyngeal carcinoma. Oncotarget.

[67] Bahrami A., Lee S., Schaefer I.M., Boland J.M., Patton K.T., Pounds S., Fletcher C.D. (2016). TERT promoter mutations and prognosis in solitary fibrous tumor. Mod. Pathol..

[68] Demicco E.G., Wani K., Ingram D., Wagner M., Maki R.G., Rizzo A., Meeker A., Lazar A.J., Wang W.L. (2018). TERT promoter mutations in solitary fibrous tumour. Histopathology.

[69] Vogels R., Macagno N., Griewank K., Groenen P., Verdijk M., Fonville J., Kusters B., Figarella-Branger D., Wesseling P., Bouvier C. (2019). Prognostic significance of NAB2-STAT6 fusion variants and TERT promotor mutations in solitary fibrous tumors/hemangiopericytomas of the CNS: Not (yet) clear. Acta Neuropathol..

[70] Chmielecki J., Crago A.M., Rosenberg M., O’Connor R., Walker S.R., Ambrogio L., Auclair D., McKenna A., Heinrich M.C., Frank D.A. (2013). Whole-exome sequencing identifies a recurrent NAB2-STAT6 fusion in solitary fibrous tumors. Nat. Genet..

[71] Robinson D.R., Wu Y.M., Kalyana-Sundaram S., Cao X., Lonigro R.J., Sung Y.S., Chen C.L., Zhang L., Wang R., Su F. (2013). Identification of recurrent NAB2-STAT6 gene fusions in solitary fibrous tumor by integrative sequencing. Nat. Genet..

[72] Mohajeri A., Tayebwa J., Collin A., Nilsson J., Magnusson L., von Steyern F.V., Brosjo O., Domanski H.A., Larsson O., Sciot R. (2013). Comprehensive genetic analysis identifies a pathognomonic NAB2/STAT6 fusion gene, nonrandom secondary genomic imbalances, and a characteristic gene expression profile in solitary fibrous tumor. Genes Chromosomes Cancer.

[73] Lai A.Y., Wade P.A. (2011). Cancer biology and NuRD: A multifaceted chromatin remodelling complex. Nat. Rev. Cancer.

[74] Reynolds N., Latos P., Hynes-Allen A., Loos R., Leaford D., O’Shaughnessy A., Mosaku O., Signolet J., Brennecke P., Kalkan T. (2012). NuRD suppresses pluripotency gene expression to promote transcriptional heterogeneity and lineage commitment. Cell Stem Cell.

[75] Li J., Rodriguez J.P., Niu F., Pu M., Wang J., Hung L.W., Shao Q., Zhu Y., Ding W., Liu Y. (2016). Structural basis for DNA recognition by STAT6. Proc. Nat. Acad. Sci. USA.

[76] Barthelmess S., Geddert H., Boltze C., Moskalev E.A., Bieg M., Sirbu H., Brors B., Wiemann S., Hartmann A., Agaimy A. (2014). Solitary fibrous tumors/hemangiopericytomas with different variants of the NAB2-STAT6 gene fusion are characterized by specific histomorphology and distinct clinicopathological features. Am. J. Pathol..

[77] Tai H.C., Chuang I.C., Chen T.C., Li C.F., Huang S.C., Kao Y.C., Lin P.C., Tsai J.W., Lan J., Yu S.C. (2015). NAB2-STAT6 fusion types account for clinicopathological variations in solitary fibrous tumors. Mod. Pathol..

[78] Bieg M., Moskalev E.A., Will R., Hebele S., Schwarzbach M., Schmeck S., Hohenberger P., Jakob J., Kasper B., Gaiser T. (2021). Gene Expression in Solitary Fibrous Tumors (SFTs) Correlates with Anatomic Localization and NAB2-STAT6 Gene Fusion Variants. Am. J. Pathol..

[79] Hatva E., Bohling T., Jaaskelainen J., Persico M.G., Haltia M., Alitalo K. (1996). Vascular growth factors and receptors in capillary hemangioblastomas and hemangiopericytomas. Am. J. Pathol..

[80] Sawada N., Ishiwata T., Naito Z., Maeda S., Sugisaki Y., Asano G. (2002). Immunohistochemical localization of endothelial cell markers in solitary fibrous tumor. Pathol. Int..

[81] Demicco E.G., Wani K., Fox P.S., Bassett R.L., Young E.D., Lev D., Aldape K.D., Lazar A.J., Wang W.L. (2015). Histologic variability in solitary fibrous tumors reflects angiogenic and growth factor signaling pathway alterations. Hum. Pathol..

[82] Silverman E.S., Khachigian L.M., Santiago F.S., Williams A.J., Lindner V., Collins T. (1999). Vascular smooth muscle cells express the transcriptional corepressor NAB2 in response to injury. Am. J. Pathol..

[83] Houston P., Campbell C.J., Svaren J., Milbrandt J., Braddock M. (2001). The transcriptional corepressor NAB2 blocks Egr-1-mediated growth factor activation and angiogenesis. Biochem. Biophys. Res. Commun..

[84] Lucerna M., Mechtcheriakova D., Kadl A., Schabbauer G., Schafer R., Gruber F., Koshelnick Y., Muller H.D., Issbrucker K., Clauss M. (2003). NAB2, a corepressor of EGR-1, inhibits vascular endothelial growth factor-mediated gene induction and angiogenic responses of endothelial cells. J. Biol. Chem..

[85] Punetha M., Chouhan V.S., Sonwane A., Singh G., Bag S., Green J.A., Whitworth K., Sarkar M. (2020). Early growth response gene mediates in VEGF and FGF signaling as dissected by CRISPR in corpus luteum of water buffalo. Sci. Rep..

[86] Jiang Z.L., Ripamonte P., Buratini J., Portela V.M., Price C.A. (2011). Fibroblast growth factor-2 regulation of Sprouty and NR4A genes in bovine ovarian granulosa cells. J. Cell. Physiol..

[87] Bae S.K., Bae M.H., Ahn M.Y., Son M.J., Lee Y.M., Bae M.K., Lee O.H., Park B.C., Kim K.W. (1999). Egr-1 mediates transcriptional activation of IGF-II gene in response to hypoxia. Cancer Res..

[88] Pierscianek D., Michel A., Hindy N.E., Keyvani K., Dammann P., Oezkan N., Mueller O., Sure U., Zhu Y. (2016). Activation of multiple angiogenic signaling pathways in hemangiopericytoma. Brain Tumor Pathol..

[89] Baetta R., Soma M., De-Fraja C., Comparato C., Teruzzi C., Magrassi L., Cattaneo E. (2000). Upregulation and activation of Stat6 precede vascular smooth muscle cell proliferation in carotid artery injury model. Arterioscler. Thromb. Vasc. Biol..

[90] Cao Q., Zhang T., Jiang L., Wang L., Luo S. (2010). Expression and significance of STAT3 and VEGF with MVD in the nasal polyps. J. Clin. Otorhinolaryngol. Head Neck Surg..

[91] Tang X., Yang Y., Yuan H., You J., Burkatovskaya M., Amar S. (2013). Novel transcriptional regulation of VEGF in inflammatory processes. J. Cell. Mol. Med..

[92] Judson I., Verweij J., Gelderblom H., Hartmann J.T., Schoffski P., Blay J.Y., Kerst J.M., Sufliarsky J., Whelan J., Hohenberger P. (2014). Doxorubicin alone versus intensified doxorubicin plus ifosfamide for first-line treatment of advanced or metastatic soft-tissue sarcoma: A randomised controlled phase 3 trial. Lancet Oncol..

[93] Ryan C.W., Merimsky O., Agulnik M., Blay J.Y., Schuetze S.M., Van Tine B.A., Jones R.L., Elias A.D., Choy E., Alcindor T. (2016). PICASSO III: A Phase III, Placebo-Controlled Study of Doxorubicin with or Without Palifosfamide in Patients With Metastatic Soft Tissue Sarcoma. J. Clin. Oncol..

[94] Tap W.D., Papai Z., Van Tine B.A., Attia S., Ganjoo K.N., Jones R.L., Schuetze S., Reed D., Chawla S.P., Riedel R.F. (2017). Doxorubicin plus evofosfamide versus doxorubicin alone in locally advanced, unresectable or metastatic soft-tissue sarcoma (TH CR-406/SARC021): An international, multicentre, open-label, randomised phase 3 trial. Lancet Oncol..

[95] Tap W.D., Wagner A.J., Schoffski P., Martin-Broto J., Krarup-Hansen A., Ganjoo K.N., Yen C.C., Abdul Razak A.R., Spira A., Kawai A. (2020). Effect of Doxorubicin Plus Olaratumab vs Doxorubicin Plus Placebo on Survival in Patients With Advanced Soft Tissue Sarcomas: The ANNOUNCE Randomized Clinical Trial. JAMA.

[96] Gewirtz D.A. (1999). A critical evaluation of the mechanisms of action proposed for the antitumor effects of the anthracycline antibiotics adriamycin and daunorubicin. Biochem. Pharmacol..

[97] Chang Y.L., Lee Y.C., Wu C.T. (1999). Thoracic solitary fibrous tumor: Clinical and pathological diversity. Lung Cancer.

[98] Harrison-Phipps K.M., Nichols F.C., Schleck C.D., Deschamps C., Cassivi S.D., Schipper P.H., Allen M.S., Wigle D.A., Pairolero P.C. (2009). Solitary fibrous tumors of the pleura: Results of surgical treatment and long-term prognosis. J. Thorac. Cardiovasc. Surg..

[99] Zhou C., Li W., Shao J., Zhao J. (2020). Thoracic solitary fibrous tumors: An analysis of 70 patients who underwent surgical resection in a single institution. J. Cancer Res. Clin. Oncol..

[100] Aridi T., Tawil A., Hashem M., Khoury J., Raad R.A., Youssef P. (2019). Unique Presentation and Management Approach of Pleural Solitary Fibrous Tumor. Case Rep. Surg..

[101] Dingley B., Fiore M., Gronchi A. (2019). Personalizing surgical margins in retroperitoneal sarcomas: An update. Expert Rev. Anticancer. Ther..

[102] Wang Y., Wei R., Ji T., Chen Z., Guo W. (2018). Surgical treatment of primary solitary fibrous tumors involving the pelvic ring. PLoS ONE.

[103] Carneiro S.S., Scheithauer B.W., Nascimento A.G., Hirose T., Davis D.H. (1996). Solitary fibrous tumor of the meninges: A lesion distinct from fibrous meningioma. A clinicopathologic and immunohistochemical study. Am. J. Clin. Pathol..

[104] Kim J.M., Choi Y.L., Kim Y.J., Park H.K. (2017). Comparison and evaluation of risk factors for meningeal, pleural, and extrapleural solitary fibrous tumors: A clinicopathological study of 92 cases confirmed by STAT6 immunohistochemical staining. Pathol. Res. Pract..

[105] Haas R.L., Walraven I., Lecointe-Artzner E., van Houdt W.J., Scholten A.N., Strauss D., Schrage Y., Hayes A.J., Raut C.P., Fairweather M. (2021). Management of meningeal solitary fibrous tumors/hemangiopericytoma; surgery alone or surgery plus postoperative radiotherapy?. Acta Oncol..

[106] Haas R.L., Walraven I., Lecointe-Artzner E., van Houdt W.J., Strauss D., Schrage Y., Hayes A.J., Raut C.P., Fairweather M., Baldini E.H. (2020). Extrameningeal solitary fibrous tumors-surgery alone or surgery plus perioperative radiotherapy: A retrospective study from the global solitary fibrous tumor initiative in collaboration with the Sarcoma Patients EuroNet. Cancer.

[107] Lee J.H., Jeon S.H., Park C.K., Park S.H., Yoon H.I., Chang J.H., Suh C.O., Kang S.J., Lim D.H., Kim I.A. (2021). The Role of Postoperative Radiotherapy in Intracranial Solitary Fibrous Tumor/Hemangiopericytoma: A Multi-Institutional Retrospective Study (KROG 18-11). Cancer Res. Treat..

[108] Krengli M., Cena T., Zilli T., Jereczek-Fossa B.A., De Bari B., Villa Freixa S., Kaanders J., Torrente S., Pasquier D., Sole C.V. (2020). Radiotherapy in the treatment of extracranial hemangiopericytoma/solitary fibrous tumor: Study from the Rare Cancer Network. Radiother. Oncol..

[109] Haas R.L., Walraven I., Lecointe-Artzner E., Scholten A.N., van Houdt W.J., Griffin A.M., Ferguson P.C., Miah A.B., Zaidi S., DeLaney T.F. (2018). Radiation Therapy as Sole Management for Solitary Fibrous Tumors (SFT): A Retrospective Study From the Global SFT Initiative in Collaboration With the Sarcoma Patients EuroNet. Int. J. Radiat. Oncol. Biol. Phys..

[110] Martin-Broto J., Hindi N., Lopez-Pousa A., Peinado-Serrano J., Alvarez R., Alvarez-Gonzalez A., Italiano A., Sargos P., Cruz-Jurado J., Isern-Verdum J. (2020). Assessment of Safety and Efficacy of Combined Trabectedin and Low-Dose Radiotherapy for Patients with Metastatic Soft-Tissue Sarcomas: A Nonrandomized Phase 1/2 Clinical Trial. JAMA Oncol..

[111] Fletcher C.D.M., Bridge J.A., Hogendoorn P.C.W., Mertens F. (2013). WHO Classification of Tumours of Soft Tissue and Bone.

[112] Stacchiotti S., Saponara M., Frapolli R., Tortoreto M., Cominetti D., Provenzano S., Negri T., Dagrada G.P., Gronchi A., Colombo C. (2017). Patient-derived solitary fibrous tumour xenografts predict high sensitivity to doxorubicin/dacarbazine combination confirmed in the clinic and highlight the potential effectiveness of trabectedin or eribulin against this tumour. Eur. J. Cancer.

[113] Stacchiotti S., Libertini M., Negri T., Palassini E., Gronchi A., Fatigoni S., Poletti P., Vincenzi B., Dei Tos A.P., Mariani L. (2013). Response to chemotherapy of solitary fibrous tumour: A retrospective study. Eur. J. Cancer.

[114] Constantinidou A., Jones R.L., Olmos D., Thway K., Fisher C., Al-Muderis O., Judson I. (2012). Conventional anthracycline-based chemotherapy has limited efficacy in solitary fibrous tumour. Acta Oncol..

[115] Levard A., Derbel O., Meeus P., Ranchere D., Ray-Coquard I., Blay J.Y., Cassier P.A. (2013). Outcome of patients with advanced solitary fibrous tumors: The Centre Leon Berard experience. BMC Cancer.

[116] Schoffski P., Timmermans I., Hompes D., Stas M., Sinnaeve F., De Leyn P., Coosemans W., Van Raemdonck D., Hauben E., Sciot R. (2020). Clinical Presentation, Natural History, and Therapeutic Approach in Patients with Solitary Fibrous Tumor: A Retrospective Analysis. Sarcoma.

[117] Park M.S., Ravi V., Conley A., Patel S.R., Trent J.C., Lev D.C., Lazar A.J., Wang W.L., Benjamin R.S., Araujo D.M. (2013). The role of chemotherapy in advanced solitary fibrous tumors: A retrospective analysis. Clin. Sarcoma Res..

[118] Outani H., Kobayashi E., Wasa J., Saito M., Takenaka S., Hayakawa K., Endo M., Takeuchi A., Kobayashi H., Kito M. (2020). Clinical Outcomes of Patients with Metastatic Solitary Fibrous Tumors: A Japanese Musculoskeletal Oncology Group (JMOG) Multiinstitutional Study. Ann. Surg. Oncol..

[119] Stacchiotti S., Tortoreto M., Bozzi F., Tamborini E., Morosi C., Messina A., Libertini M., Palassini E., Cominetti D., Negri T. (2013). Dacarbazine in solitary fibrous tumor: A case series analysis and preclinical evidence vis-a-vis temozolomide and antiangiogenics. Clin. Cancer Res..

[120] Chaigneau L., Kalbacher E., Thiery-Vuillemin A., Fagnoni-Legat C., Isambert N., Aherfi L., Pauchot J., Delroeux D., Servagi-Vernat S., Mansi L. (2011). Efficacy of trabectedin in metastatic solitary fibrous tumor. Rare Tumors.

[121] Khalifa J., Ouali M., Chaltiel L., Le Guellec S., Le Cesne A., Blay J.Y., Cousin P., Chaigneau L., Bompas E., Piperno-Neumann S. (2015). Efficacy of trabectedin in malignant solitary fibrous tumors: A retrospective analysis from the French Sarcoma Group. BMC Cancer.

[122] Kobayashi H., Iwata S., Wakamatsu T., Hayakawa K., Yonemoto T., Wasa J., Oka H., Ueda T., Tanaka S. (2020). Efficacy and safety of trabectedin for patients with unresectable and relapsed soft-tissue sarcoma in Japan: A Japanese Musculoskeletal Oncology Group study. Cancer.

[123] Park M.S., Patel S.R., Ludwig J.A., Trent J.C., Conrad C.A., Lazar A.J., Wang W.L., Boonsirikamchai P., Choi H., Wang X. (2011). Activity of temozolomide and bevacizumab in the treatment of locally advanced, recurrent, and metastatic hemangiopericytoma and malignant solitary fibrous tumor. Cancer.

[124] Kawai A., Araki N., Sugiura H., Ueda T., Yonemoto T., Takahashi M., Morioka H., Hiraga H., Hiruma T., Kunisada T. (2015). Trabectedin monotherapy after standard chemotherapy versus best supportive care in patients with advanced, translocation-related sarcoma: A randomised, open-label, phase 2 study. Lancet Oncol..

[125] Forni C., Minuzzo M., Virdis E., Tamborini E., Simone M., Tavecchio M., Erba E., Grosso F., Gronchi A., Aman P. (2009). Trabectedin (ET-743) promotes differentiation in myxoid liposarcoma tumors. Mol. Cancer Ther..

[126] Grohar P.J., Segars L.E., Yeung C., Pommier Y., D’Incalci M., Mendoza A., Helman L.J. (2014). Dual targeting of EWS-FLI1 activity and the associated DNA damage response with trabectedin and SN38 synergistically inhibits Ewing sarcoma cell growth. Clin. Cancer Res..

[127] Schoffski P., Chawla S., Maki R.G., Italiano A., Gelderblom H., Choy E., Grignani G., Camargo V., Bauer S., Rha S.Y. (2016). Eribulin versus dacarbazine in previously treated patients with advanced liposarcoma or leiomyosarcoma: A randomised, open-label, multicentre, phase 3 trial. Lancet.

[128] Schoffski P., Ray-Coquard I.L., Cioffi A., Bui N.B., Bauer S., Hartmann J.T., Krarup-Hansen A., Grunwald V., Sciot R., Dumez H. (2011). Activity of eribulin mesylate in patients with soft-tissue sarcoma: A phase 2 study in four independent histological subtypes. Lancet Oncol..

[129] Kawai A., Araki N., Naito Y., Ozaki T., Sugiura H., Yazawa Y., Morioka H., Matsumine A., Saito K., Asami S. (2017). Phase 2 study of eribulin in patients with previously treated advanced or metastatic soft tissue sarcoma. Jpn. J. Clin. Oncol..

[130] Stacchiotti S., Negri T., Libertini M., Palassini E., Marrari A., De Troia B., Gronchi A., Dei Tos A.P., Morosi C., Messina A. (2012). Sunitinib malate in solitary fibrous tumor (SFT). Ann. Oncol..

[131] Maruzzo M., Martin-Liberal J., Messiou C., Miah A., Thway K., Alvarado R., Judson I., Benson C. (2015). Pazopanib as first line treatment for solitary fibrous tumours: The Royal Marsden Hospital experience. Clin. Sarcoma Res..

[132] Mulamalla K., Truskinovsky A.M., Dudek A.Z. (2008). Rare case of hemangiopericytoma responds to sunitinib. Transl. Res..

[133] George S., Merriam P., Maki R.G., Van den Abbeele A.D., Yap J.T., Akhurst T., Harmon D.C., Bhuchar G., O’Mara M.M., D’Adamo D.R. (2009). Multicenter phase II trial of sunitinib in the treatment of nongastrointestinal stromal tumor sarcomas. J. Clin. Oncol..

[134] Domont J., Massard C., Lassau N., Armand J.P., Le Cesne A., Soria J.C. (2010). Hemangiopericytoma and antiangiogenic therapy: Clinical benefit of antiangiogenic therapy (sorafenib and sunitinib) in relapsed malignant haemangioperyctoma /solitary fibrous tumour. Investig. New Drugs.

[135] Martin-Broto J., Hindi N., Grignani G., Martinez-Trufero J., Redondo A., Valverde C., Stacchiotti S., Lopez-Pousa A., D’Ambrosio L., Gutierrez A. (2020). Nivolumab and sunitinib combination in advanced soft tissue sarcomas: A multicenter, single-arm, phase Ib/II trial. J. Immunother. Cancer.

[136] Valentin T., Fournier C., Penel N., Bompas E., Chaigneau L., Isambert N., Chevreau C. (2013). Sorafenib in patients with progressive malignant solitary fibrous tumors: A subgroup analysis from a phase II study of the French Sarcoma Group (GSF/GETO). Investig. New Drugs.

[137] Xie C., Wan X., Quan H., Zheng M., Fu L., Li Y., Lou L. (2018). Preclinical characterization of anlotinib, a highly potent and selective vascular endothelial growth factor receptor-2 inhibitor. Cancer Sci..

[138] Hu-Lowe D.D., Zou H.Y., Grazzini M.L., Hallin M.E., Wickman G.R., Amundson K., Chen J.H., Rewolinski D.A., Yamazaki S., Wu E.Y. (2008). Nonclinical antiangiogenesis and antitumor activities of axitinib (AG-013736), an oral, potent, and selective inhibitor of vascular endothelial growth factor receptor tyrosine kinases 1, 2, 3. Clin. Cancer Res..

[139] You W.K., Sennino B., Williamson C.W., Falcón B., Hashizume H., Yao L.C., Aftab D.T., McDonald D.M. (2011). VEGF and c-Met blockade amplify angiogenesis inhibition in pancreatic islet cancer. Cancer Res..

[140] Matsui J., Yamamoto Y., Funahashi Y., Tsuruoka A., Watanabe T., Wakabayashi T., Uenaka T., Asada M. (2008). E7080, a novel inhibitor that targets multiple kinases, has potent antitumor activities against stem cell factor producing human small cell lung cancer H146, based on angiogenesis inhibition. Int. J. Cancer.

[141] Harris P.A., Boloor A., Cheung M., Kumar R., Crosby R.M., Davis-Ward R.G., Epperly A.H., Hinkle K.W., Hunter R.N., Johnson J.H. (2008). Discovery of 5-[[4-[(2,3-dimethyl-2H-indazol-6-yl)methylamino]-2-pyrimidinyl]amino]-2-methyl-benzenesulfonamide (Pazopanib), a novel and potent vascular endothelial growth factor receptor inhibitor. J. Med. Chem..

[142] Wilhelm S.M., Dumas J., Adnane L., Lynch M., Carter C.A., Schütz G., Thierauch K.H., Zopf D. (2011). Regorafenib (BAY 73-4506): A new oral multikinase inhibitor of angiogenic, stromal and oncogenic receptor tyrosine kinases with potent preclinical antitumor activity. Int. J. Cancer.

[143] Wilhelm S.M., Carter C., Tang L., Wilkie D., McNabola A., Rong H., Chen C., Zhang X., Vincent P., McHugh M. (2004). BAY 43-9006 exhibits broad spectrum oral antitumor activity and targets the RAF/MEK/ERK pathway and receptor tyrosine kinases involved in tumor progression and angiogenesis. Cancer Res..

[144] Chow L.Q.M., Eckhardt S.G. (2007). Sunitinib: From Rational Design to Clinical Efficacy. J. Clin. Oncol..

[145] Gitenay D., Baron V.T. (2009). Is EGR1 a potential target for prostate cancer therapy?. Future Oncol..

[146] Wang W.L., Gokgoz N., Samman B., Andrulis I.L., Wunder J.S., Demicco E.G. (2020). RNA expression profiling reveals PRAME, a potential immunotherapy target, is frequently expressed in solitary fibrous tumors. Mod. Pathol..

[147] Chalmers Z.R., Connelly C.F., Fabrizio D., Gay L., Ali S.M., Ennis R., Schrock A., Campbell B., Shlien A., Chmielecki J. (2017). Analysis of 100,000 human cancer genomes reveals the landscape of tumor mutational burden. Genome Med..

[148] Ludwig J.A., Federman N., Anderson P., Macy M.E., Davis L.E., Riedel R.F., Muscal J.A., Daw N.C., Ratan R., Toretsky J. (2020). Phase I study of TK216, a novel anti-ETS agent for Ewing sarcoma. Ann. Oncol..

[149] Huijbers E.J.M., van der Werf I.M., Faber L.D., Sialino L.D., van der Laan P., Holland H.A., Cimpean A.M., Thijssen V., van Beijnum J.R., Griffioen A.W. (2019). Targeting Tumor Vascular CD99 Inhibits Tumor Growth. Front. Immunol..

